# Nonmechanistic forecasts of seasonal influenza with iterative one-week-ahead distributions

**DOI:** 10.1371/journal.pcbi.1006134

**Published:** 2018-06-15

**Authors:** Logan C. Brooks, David C. Farrow, Sangwon Hyun, Ryan J. Tibshirani, Roni Rosenfeld

**Affiliations:** 1 School of Computer Science, Carnegie Mellon University, Pittsburgh, Pennsylvania, United States of America; 2 Department of Statistics, Carnegie Mellon University, Pittsburgh, Pennsylvania, United States of America; National Institutes of Health, UNITED STATES

## Abstract

Accurate and reliable forecasts of seasonal epidemics of infectious disease can assist in the design of countermeasures and increase public awareness and preparedness. This article describes two main contributions we made recently toward this goal: a novel approach to probabilistic modeling of surveillance time series based on “delta densities”, and an optimization scheme for combining output from multiple forecasting methods into an adaptively weighted ensemble. Delta densities describe the probability distribution of the change between one observation and the next, conditioned on available data; chaining together nonparametric estimates of these distributions yields a model for an entire trajectory. Corresponding distributional forecasts cover more observed events than alternatives that treat the whole season as a unit, and improve upon multiple evaluation metrics when extracting key targets of interest to public health officials. Adaptively weighted ensembles integrate the results of multiple forecasting methods, such as delta density, using weights that can change from situation to situation. We treat selection of optimal weightings across forecasting methods as a separate estimation task, and describe an estimation procedure based on optimizing cross-validation performance. We consider some details of the data generation process, including data revisions and holiday effects, both in the construction of these forecasting methods and when performing retrospective evaluation. The delta density method and an adaptively weighted ensemble of other forecasting methods each improve significantly on the next best ensemble component when applied separately, and achieve even better cross-validated performance when used in conjunction. We submitted real-time forecasts based on these contributions as part of CDC’s 2015/2016 FluSight Collaborative Comparison. Among the fourteen submissions that season, this system was ranked by CDC as the most accurate.

## Author summary

Seasonal influenza is associated with 250 000 to 500 000 deaths worldwide each year (WHO estimates). In the United States and other temperate regions, seasonal influenza epidemics occur annually, but their timing and intensity varies significantly; accurate and reliable forecasts that quantify their uncertainty can assist policymakers when planning countermeasures such as vaccination campaigns, and increase awareness and preparedness of hospitals and the general public. Starting with the 2013/2014 flu season, CDC has solicited, collected, evaluated, and compared weekly forecasts from external research groups. We developed a new method for forecasting flu surveillance data, which stitches together models of changes that happen each week, and a way of combining its output with other forecasts. The resulting forecasting system produced the most accurate forecasts in CDC’s 2015/2016 FluSight comparison of fourteen forecasting systems. We describe our new forecasting methods, analyze their performance in the 2015/2016 comparison and on data from previous seasons, and describe idiosyncrasies of epidemiological data that should be considered when constructing and evaluating forecasting systems.

## Introduction

Seasonal influenza epidemics cause widespread illness which is associated each year with an estimated 250 000 to 500 000 deaths worldwide [1] and 3000 to 56 000 deaths in the United States alone [2–4]. In contrast to influenza “pandemics”, which are rare global outbreaks of especially novel influenza A viruses [5, 6], seasonal epidemics (i.e., non-pandemics), while still having worldwide reach, occur annually in the United States and other countries with (generally) temperate climates. Time series of influenza prevalence in these areas are typically low and flat for the majority of the season, but trace a single, sharp peak sometime during winter, with significant variability in timing and intensity. Accurate and reliable forecasts of seasonal epidemics can help policymakers plan countermeasures such as vaccination campaigns, and increase awareness and preparedness of hospitals and the general public. The Centers for Disease Control and Prevention (CDC) monitors influenza prevalence with several well-established surveillance systems [7]; the recurring nature of seasonal epidemics and availability of historical data provide promising opportunities for the formation, evaluation, and application of statistical models. Starting with the 2013/2014 “Predict the Influenza Season Challenge” [8] and continuing each season thereafter as the Epidemic Prediction Initiative’s FluSight project [9], CDC has solicited and compiled forecasts of influenza-like illness (ILI) prevalence from external research groups and worked with them to develop standardized forecast formats and quantitative evaluation metrics.

Various approaches to influenza epidemic forecasting are summarized in literature reviews [10–12] and descriptions of the CDC comparisons [8, 9]. Some common approaches are described below, with references to work applicable to the current FluSight project and related seasonal dengue forecasting tasks, emphasizing more recent work that may not be listed in the above three literature reviews:

- **Mechanistic models**: describe the disease state and interaction between individuals with causal models, as well as the surveillance data generation process.
  - **Compartmental models** (e.g., [13–17]): break down the population into a number of discrete “compartments” describing their characteristics (e.g., age, location) and state (e.g., susceptible to, infectious with, or recovered from a particular disease), and describe how the occupancy of these compartments changes over time, either deterministically or probabilistically. In many of these models, this division describes solely the state with respect to a single disease, ignoring details regarding age, spatial dynamics, and mixtures of ILI diseases, but keeping the number of parameters to infer low.
  - **Agent-based models** (e.g., [11, 18]): also known as individual-based models, these approaches use more detailed descriptions of disease state and/or individual characteristics and behavior, which are not easily simplified into a compartmental form, typically studied using computation-heavy simulations. These approaches usually include many more parameters than compartmental models, which may be set or inferred by heuristics, additional data sources and studies, or Monte Carlo procedures.
- **Phenomenological models**: also referred to as statistical models, these approaches describe the surveillance data without directly incorporating the epidemiological underpinnings.
  - **Direct regression models** (e.g., [19–22]): attempt to estimate future prevalence or targets of interest using various types of regression, including nonparametric statistical approaches and alternatives from machine learning literature.
  - **Time series models** (e.g., [23–31]): represent the expected value of (transformations of) observations and/or underlying latent state at a particular time as (typically linear) functions of these quantities at previous times and additional covariates, paired with Gaussian, Poisson, negative binomial, or other noise distributions. This category includes linear dynamical systems and frameworks such as SARIMAX.

We present a novel phenomenological approach to epidemiological forecasting using “delta densities”, which assumes an autoregressive dependency structure similar to those of some time series models, but uses a kernel density estimation approach to model these dependencies rather than the common choice of linear relationships plus Gaussian noise. This technique is similar to the method of analogues [19] in that it uses an instance-based, nonparametric estimation procedure, but provides distributional forecasts of entire trajectories rather than point predictions of individual observations. The kernel conditional density estimation (KCDE) forecasting method [22] attacks many of the same issues encountered when applying kernel density estimation methods to seasonal epidemic data, but models the dependency structure of future weeks with a copula, while delta density chains together 1-week-ahead simulations. Compared to approaches that treat the entire season as a unit, such as deterministic, single-strain, fully-mixed compartmental models [13] or our previous empirical Bayes approach based on modifying past seasons’ data [21, 32], this method forms a larger library of possible trajectories by piecing together local models, which appears to help forecast performance, even though the trajectories considered may seem less reasonable on average.

Our second contribution is an adaptively weighted ensemble approach to combining the output of different forecasting methods given their historical and/or cross-validation forecasts. We first implemented this method in preparation for the 2014/2015 FluSight comparison, mixing together our empirical Bayes forecasting method with two baselines (a uniform distribution and an empirical distribution for each target), and later applied it while participating in the Dengue Forecasting project [33] and following FluSight comparisons (adding up to 9 additional components including delta density based methods), and found it improved our forecasts in all cases. Other epidemic forecasting teams have also reported success with concurrently or subsequently developed stacking generalization [34, 35] ensemble approaches to the FluSight forecasting tasks using Bayesian model averaging [36], the fixed weighting scheme that we examine below [37], and alternative adaptive weighting schemes based on gradient tree boosting [37], as well as with earlier ensemble approaches to short-term point predictions [20]. Methodologically, our adaptively weighted ensemble framework differs from these alternatives in that it selects a weighting over components for a particular forecast using “plug-in” statistical estimators for the optimal weights given the context of the forecast being prepared. Like the adaptive approaches presented in [37], component weights for each forecast are selected using regression, but the type of regression used and the manner of incorporating additional information, such as the forecast week, are distinct.

## Materials and methods

### Surveillance data

Recording every case of influenza is not practicable; infections are often asymptomatic [38] or symptomatic but not clinically attended [39], laboratory testing may not be performed for clinically attended cases or give false negative results, and reporting of lab-confirmed cases is not mandatory in most instances. Forecast comparisons are instead based on syndromic clinical surveillance data from the U.S. Outpatient Influenza-like Illness Surveillance Network (ILINet) [7, 40], a group of health care providers that voluntarily report statistics regarding ILI, where ILI is defined as a 100°F (37.8°C) fever with a cough and/or sore throat without a known cause other than influenza. CDC aggregates these reports and estimates the weekly percentage of patients seen that have ILI, %ILI, across all health care providers using a measure called weighted %ILI (wILI).

- **Geographical resolution**: CDC reports wILI for each of the 10 U.S. Department of Health & Human Services (HHS) regions, as well as for the nation as a whole; the wILI for each of these locations is a weighted average of the ILINet %ILI for state-level units based on population.
- **Temporal resolution**: wILI is available on a weekly basis; weeks begin on Sunday, end on Saturday, and are numbered according to the epidemiological week (epi week) convention in the United States.
- **Timeliness**: Initial wILI estimates for a given week are typically released on Friday of the following week; additional reports and revisions from participating health care providers are incorporated in updates throughout the season and after it ends.
- **Specificity**: Influenza is just one of many potential causes of ILI. Laboratory testing data [7] suggest that influenza is responsible for a significant portion of ILI cases during the flu season, especially for weeks when wILI is high, but only for a very small fraction of cases in the typical flu off-season. Much of the variance and “peakiness” in wILI can be associated with influenza epidemics, but wILI trajectories do not taper off to near-zero values as one might expect in a direct measurement of influenza prevalence.
- **Influence of non-ILI cases**: Since wILI depends on records of both ILI cases and total cases, patterns in non-ILI cases can impact wILI trajectories. We discuss one such pattern in the Holiday effects section.

CDC hosts the latest ILINet report and other types of surveillance data through Fluview Interactive, a collection of web modules [41]; we provide current and historical ILINet reports and some other data sources through our delphi-epidata API [42] and epivis visualizer [43].

### Forecasting targets

The FluSight project focuses on in-season distributional forecasts and point predictions of key targets of interest to public health officials:

- **Short-term wILI**: the four wILI values following the last available observation (incorporating all data revisions through some week well after the season’s end)
- **Season onset**: the first week in the first run of at least three consecutive weeks with wILI values above a location- and season- specific baseline wILI level set by CDC [7], or “none” if no such runs exist; describes whether and when an influenza epidemic started in a given season
- **Season peak percentage**: the maximum of all wILI values for a given season
- **Season peak week**: the week or weeks in which wILI takes on its maximum value, or “none” if there was no onset in the 2015/2016 comparison

When making distributional forecasts, wILI values are discretized into CDC-specified bins and a probability assigned to each bin, forming a histogram over possible observations. The width of the bins was set at 0.5 %wILI for the 2015/2016 comparison and 0.1 %wILI for the 2016/2017 comparison; we use a width of 0.5 %wILI for analysis of the 2015/2016 comparison prospective forecasts, and a width of 0.1 %wILI for retrospective evaluation. CDC typically presents wILI values rounded to a resolution of 0.1 %wILI; some targets and evaluations are based on these rounded values.

### Evaluation metrics

We focus on three metrics for evaluating performance of a forecast for a given target:

- **Unibin log score**: $\log{\hat{p}}_{i}$, where ${\hat{p}}_{i}$ is the probability assigned to *i*, the bin containing the observed value. We use this score for ensemble weight selection and most internal evaluation as it has ties to maximum likelihood estimation, and is “proper score” [44]. A score for a (reported) distributional prediction $\hat{p}$ is called “proper” if its expected value according to any (internal) distributional prediction $\hat{q}$ is maximized when reporting $\hat{p}=\hat{q}$, i.e., forecasters can maximize their expected scores by reporting their true beliefs. We refer to the “unibin log score” simply as the “log score” except for when comparing it with the multibin log score, which is defined next. The exponentiated mean log score is the (geometric) average probability assigned to events that were actually observed. The exponentiated difference in the mean log scores of method A and method B is an estimate of the (geometric) expected winnings of unit-sized bets of the form “this bin will hold the true value” when bets are placed optimally according to the forecasts of A, and (relative) prices are set optimally according to the forecasts of B.
- **Multibin log score**: $\log\sum_{i\;\text{near}\;\text{observed}\;\text{value}}{\hat{p}}_{i}$, where the *i*’s considered are typically bins within 0.5 %wILI of observed values for a wILI target, or within 1 week for a timing target. The multibin log score was designed by FluSight hosts in consultation with participants, and the judgment “near observed value” was selected as a level of error that would not significantly impact policymakers’ decisions. The exponentiated mean multibin log score is the (geometric) average amount of mass a forecaster placed within this margin for error of observed target values.
- **Absolute error**: $\left|\hat{y}-y\right|$, where $\hat{y}$ is the point prediction and *y* is an observed value. (In the case of onset, we consider point predictions for the value of onset conditioned on the fact that an onset actually occurs. We do not consider absolute error for onset in instances where no onset occurred. Some methods considered would sometimes fail to produce such conditional onset point predictions when they were confident that there was no onset, but these methods are not included in any of the figures containing absolute errors.)

The FluSight 2015/2016 forecast comparison evaluations were based solely on the multibin log score [45].

### Terminology and notation

The “flu season” is typically defined as epi week 40 of one year through 20 of the next; we also include data from the rest of the year as part of the season for the purpose of fitting models. In all mathematical notation, we will number the first week of the season as 1 rather than using the corresponding epi week. Let

- $W_{1..t}^{t}$ denote the *t*-th CDC report of the current season, containing wILI values for weeks 1 through *t*, inclusive, which is normally published on Friday of week *t* + 1;
- *T* be the number of weeks in the current season (either 52 or 53); we omit all details regarding differing season lengths, presenting forecasting methods and labeling epi week plot axes as if all seasons were of length *T*;
- *Y*_1..*T*_ be the ground truth wILI for the current season: the wILI values used for forecast evaluation, specifically the epi week 28 report for the FluSight comparison, or later revisions as they are available for cross-validation analysis;
- $Y_{1..T}^{s}$ be the ground truth wILI for past season *s*; and
- **Z**^*t*^ be a vector containing the forecasting targets of interest at the *t*-th wILI report of the current season: *Y*_*t*+1..*t*+4_ and the seasonal onset, peak week, and peak percentage; for the FluSight comparison, forecasts for these targets were typically due on Monday of week *t* + 2, and allowed to use ILINet and any other data released before the deadline.

Our goal is to forecast **Z**^*t*^ given $W_{1..t}^{t}$ and previous reports. This can be broken down into multiple steps, such as:

1. “Backcast” updates to the data through time *t*, producing a distribution over *Y*_1..*t*_ based on the value of $W_{1..t}^{t}$ and previous reports.
2. Connect the backcast for *Y*_1..*t*_ with corresponding forecasts for *Y*_*t*+1..*T*_, yielding a distribution for the entire trajectory *Y*_1..*t*_.
3. Calculate the distribution for **Z**^*t*^ corresponding to this distribution over *Y*_1..*t*_.

We first introduce the delta density method, which forecasts *Y*_*t*+1..*T*_ given *Y*_1..*t*_ (step 2). We then discuss a separate procedure for combining multiple forecasts into an adaptively weighted ensemble, forecasting **Z**^*t*^ given either *Y*_1..*t*_ or $W_{1..t}^{t}$ (steps 2–3 or 1–3). We also outline a method for estimating the distribution of *Y*_1..*t*_ given $W_{1..t}^{t}$ (step 1), and analyze its performance when used in conjunction with the delta density method.

### Delta density method

Consider the task of estimating the density function $f_{Y_{t+1..T}^{}|Y_{1..t}^{}}$ using an instance-based approach. Kernel density estimation and kernel regression use smoothing kernels to produce flexible estimates of the density of a random variable (e.g., $f_{Y_{t+1..T}^{}}$) and the conditional expectation of one random variable given the value of another (e.g., $E[Y_{t+1..T}^{}|Y_{1..t}^{}]$), respectively; we can combine these two methods to obtain estimates of the conditional density of one random variable given another. One possible approach would be to use the straightforward estimate

f^Yt+1..T∣Y1..t(yt+1..T∣y1..t)=∑s=1SI1..t(y1..t,Y1..ts)Ot+1..T(yt+1..T,Yt+1..Ts)∑s=1SI1..t(y1..t,Y1..ts)Ot+1..t(yt+1..t,Yt+1..ts),

where {1..*S*} is the set of fully observed historical training seasons, and *I*^1..*t*^ and *O*^*t*+1..*T*^ are smoothing kernels describing similarity between “input” trajectories and between “output” trajectories, respectively. However, while basic kernel smoothing methods can excel in low-dimensional settings, their performance scales very poorly with growing dimensionality. During most of the season, neither *Y*_1..*t*_ nor *Y*_*t*+1..*T*_ is low-dimensional, and the current season’s observations are extremely unlikely to closely match any past $Y_{1..t}^{s}$ or $Y_{t+1..T}^{s}$. This, in turn, can lead to kernel density estimates for *Y*_*t*+1..*T*_ based almost entirely on the single season *s* with the closest $Y_{1..t}^{s}$ when conditioning on *Y*_1..*t*_, and excessively narrow density estimates for *Y*_*t*+1..*T*_ even without conditioning on *Y*_1..*t*_. So, instead of applying kernel density estimation directly, we first break the task down into a sequence of low-dimensional sub-tasks. We avoid the high-dimensional output problem by chaining together estimates of $f_{\Delta Y_{u}^{}|Y_{1..u-1}^{}}$ for each *u* from *t* + 1 to *T*, where Δ*Y_u_* = *Y_u_* − *Y*_*u*−1_; estimating these single-dimensional densities requires relatively little data. However, this reformulation exacerbates the high-dimensional input problem since we are conditioning on *Y*_1..*u*−1_, which can be considerably longer than *Y*_1..*t*_. We address the high-dimensional input problem by approximating $f_{\Delta Y_{u}^{}|Y_{1..u-1}^{}}$ with $f_{\Delta Y_{u}^{}|R^{u}}$, where **R**^*u*^ is some low-dimensional vector of features derived from *Y*_1..*u*−1_. Smoothing kernel methods are used to approximate the conditional density functions using data from past seasons.

We use two sets of choices for the approximate conditional density function and summary features to form two versions of the method.

**Fig 1:**
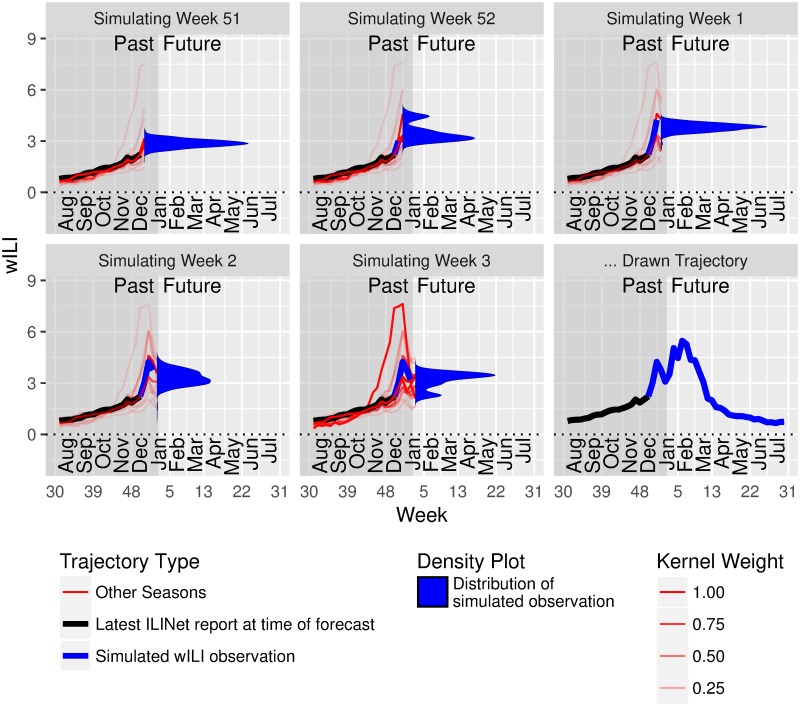
The delta density method conditions on real and simulated observations up to week *u* − 1 when building a probability distribution over the observation at week *u*. This figure demonstrates the process for drawing a single trajectory from the Markovian delta density estimate, ignoring the data revision process. The latest ILINet report, $W_{1..t}^{t}$, which incorporates observations through week 48, is shown in black. Kernel smoothing estimates for future values at times *u* from *t* + 1 to *T* are shown in blue, as are simulated observations drawn from these estimates. Past seasons’ trajectories are shown in red, with alpha values proportional to the weight they are assigned by the kernel *I*^*u*^.

- **Markovian delta density**: approximates the conditional density of Δ*Y_u_* given *Y*_1..*u*−1_ with its conditional density given just the previous (real or simulated) observation, *Y_u_*:
  f^Yt+1..T∣Y1..t(yt+1..T∣y1..t)=∏u=t+1Tf^ΔYu∣Y1..u-1(Δyu∣y1..u-1)=∏u=t+1Tf^ΔYu∣Yu-1(Δyu∣yu-1)=∏u=t+1T∑sIu(yu-1,Yu-1s)·Ou(Δyu,ΔYus)∑sIu(yu-1,Yu-1s)·Ou(Δyu,ΔYus),
  where *I*^*u*^ and *O*^*u*^ are Gaussian smoothing kernels. The first equality corresponds to the chain rule of probability on the actual (not estimated) densities; the second incorporates the Markov assumption (i.e., selects **R**^*u*^ = [*Y*_*u*−1_]); and the third gives our choice of estimators for the conditional densities ${\hat{f}}_{\Delta Y_{u}^{}|Y_{u-1}^{}}$ for each *u*. The bandwidth of each *I*^*u*^ and *O*^*u*^ is chosen separately using bandwidth selection procedures for regular kernel density estimation of *Y*_*u*−1_ and Δ*Y_u_*, respectively. (Specifically, we use the bw.SJ function from the R [46] built-in stats package, with bw.nrd0 as a fallback in the case of errors. These functions do not accept weights for the inputs; it may be possible to improve forecast performance by incorporating these weights or by using other approaches to select the bandwidths.) Note that density estimates for Δ*Y_u_* are based on data from past seasons on week *u* only, allowing the method to incorporate seasonality and holiday effects (for holidays that consistently occur at the same time of year).
  Forecasts are based on Monte Carlo simulations of *Y*_*t*+1..*T*_ ∣ *Y*_1..*t*_. The following procedure draws a single possibility for *Y*_*t*+1..*T*_:
  1. Draw $\Delta Y_{t+1}^{\text{sim}}$ from its conditional density estimate given *Y*_1..*t*_.
  2. Draw $\Delta Y_{t+2}^{\text{sim}}$ from its conditional density estimate given *Y*_1..*t*_ and $Y_{t+1}^{}=Y_{t}^{}+\Delta Y_{t+1}^{\text{sim}}$.
  3. Continue in the same manner for all remaining weeks.
  This process is illustrated in Fig 1. Repeating this procedure many times yields a sample from the model for *Y*_*t*+1..*T*_ ∣ *Y*_1..*t*_; stopping at 2000 draws seems sufficient for use in our ensemble forecasts, while at least 7000 are needed to smooth out noise when displaying distributional target forecasts for the delta density method in isolation. Any negative simulated wILI values in these trajectories are clipped off and replaced with zeroes.
- **Extended delta density**: approximates the conditional density of Δ*Y_u_* given *Y*_1..*u* − 1_ with its conditional density given four features:
  - the previous wILI value, *Y*_*u*−1_;
  - the sum of the previous *k*^*u*^ wILI values, roughly corresponding to the sum of wILI values for the current season;
  - an exponentially weighted sum of the previous *k*^*u*^ wILI; values, where the weight assigned to time *v* is 0.5^*t*′−*v*^; and
  - the previous change in wILI value, Δ*Y*_*u*−1_.
  The approximate conditional density assigns each of these features a weight (0.5, 0.25, 0.25, and 0.5, respectively) in order to reduce overfitting and emphasize some relative to the others, and incorporates data from other weeks close to *u* (specifically, within*l*^*u*^ weeks; the choice of *l*^*u*^ is discussed in a later section) with a truncated Laplacian kernel. We selected these weights and other settings, such as kernel bandwidth selection rules, somewhat arbitrarily based on intuition and experimentation on out-of-sample data; a cross-validation subroutine could be used to make the selection as well, but would multiply the amount of computation required. In case the resulting product of Gaussian and Laplacian kernels is too narrow, we mix its results with a wide boxcar kernel which evenly weights all data from time *u* − *l*^*u*^ to *u* + *l*^*u*^:
  $$\begin{array}{cc} & {\hat{f}}_{\Delta Y_{u}^{}|Y_{1..u-1}^{}}\left(\Delta y_{u}^{}|y_{1..u-1}^{}\right)\\ & =0.9\cdot \frac{\sum_{s}\sum_{v=u-l^{u}}^{u+l^{u}}0.7^{\left|v-u\right|}{\left[I_{1}^{u}\left(y_{u-1}^{},Y_{v-1}^{s}\right)\right]}^{0.5}\cdots O^{u}\left(\Delta y_{u}^{},\Delta Y_{v}^{s}\right)}{\sum_{s}\sum_{v=u-l^{u}}^{l^{u}}0.7^{\left|v-u\right|}{\left[I_{1}^{u}\left(y_{u-1}^{},Y_{v-1}^{s}\right)\right]}^{0.5}\cdots {\left[I_{4}^{u}\left(\Delta y_{u-1}^{},\Delta Y_{v-1}^{s}\right)\right]}^{0.5}}\\ & +\;0.1\cdot \frac{\sum_{s}\sum_{v=u-l^{u}}^{u+l^{u}}O^{u}\left(\Delta y_{u}^{},\Delta Y_{v}^{s}\right)}{\sum_{s}\sum_{v=u-l^{u}}^{u+l^{u}}1}. \end{array}$$
  Using data from *v* ≠ *u* incorporates additional reasonable outcomes for Δ*Y_u_* by incorporating past wILI patterns with different timing, but risks including some very unreasonable possibilities produced by repeatedly drawing from the same *v* rather than following seasonal trends with increasing *v*’s. For example, when a portion of a past season that is more similar to itself with a slight time shift than to any other past season, it may be selected for multiple consecutive *u*’s and produce an unreasonable trajectory. This could potentially occur when drawing data from the relatively flat regions of wILI trajectories of many seasons, or when incorporating observations around an unusually early, late, high, or low peak. To prevent this possibility, we combine the natural estimate for *Y_u_* arising from the density estimate for Δ*Y_u_* with a random draw $Y_{u}^{\text{uncond}}$ from the unconditional density estimate for *Y_u_* (using a Gaussian kernel and only data from week *u*):
  $$\begin{array}{c} Y_{u}^{\text{sim}}=0.9\cdot \left(Y_{u-1}^{}+\Delta Y_{u}^{\text{sim}}\right)+0.1\cdot Y_{u}^{\text{uncond}}. \end{array}$$

### Residual density method

This same approach can be applied to estimate the distribution of residuals of a wILI point predictor. Suppose that we have observed

- $Y_{1..t_{1}}^{}$, the partial wILI trajectory up to time *t*_1_, and
- $X_{1..t_{2}}^{}$, point estimates of the trajectory up to some later time *t*_2_;

our goal is to estimate the conditional distribution of

- ${\left(Y-X\right)}_{t_{1}+1..t_{2}}^{}$, the unknown residuals,

given $Y_{1..t_{1}}^{}$ and $X_{1..t_{2}}^{}$, using data from past seasons. This can be achieved by chaining together draws from conditional density estimates of (*Y* − *X*)_*u*_ ∣ **R**^*u*^ for *u* from *t*_1_ + 1 to *t*_2_, where **R**^*u*^ is a function of *Y*_1..*u*−1_ and $X_{1..t_{2}}^{}$. The delta density method can be seen as a special case where *t*_1_ = *t*; *t*_2_ = *T*; *X*_1..*t*_ = *Y*_1..*t*_, past values of *Y* which are treated as known and are simply duplicated in the simulated trajectories; and *X*_*t*+1..*T*_ = *Y*_*t..T*−1_, values of *Y* which begin as unknown but are filled in as needed by previous simulation steps, giving (*Y* − *X*)_*t*+1..*T*_ = Δ*Y*_*t*+1..*T*_. We use the residual density method to backcast *Y*_1..*t*_ from $W_{1..t}^{t}$ and as the basis for another forecaster in the ensemble.

**Fig 2:**
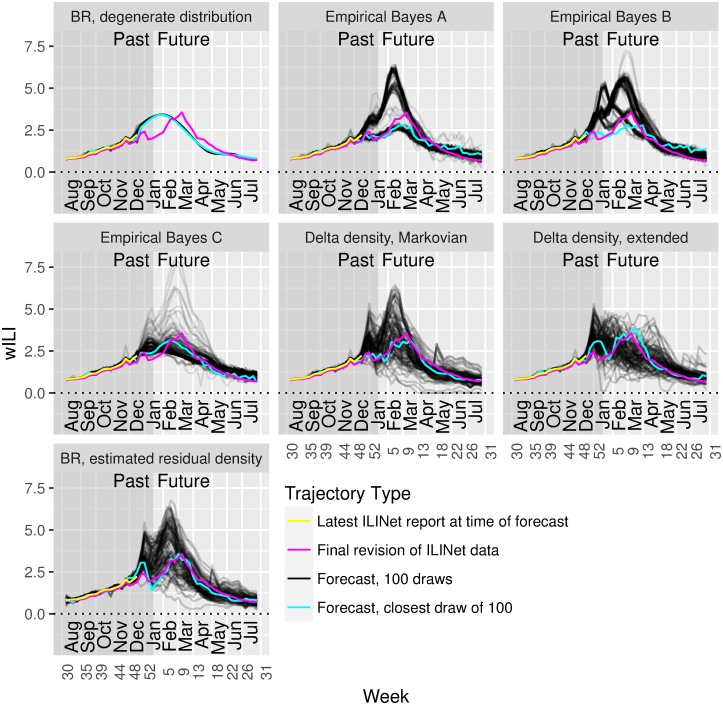
Delta and residual density methods generate wider distributions over trajectories than methods that treat entire seasons as units. These plots show sample forecasts of wILI trajectories generated from models that treat seasons as units (BR, Empirical Bayes) and from models incorporating delta and residual density methods. Yellow, the latest wILI report available for these forecasts; magenta, the ground truth wILI available at the beginning of the following season; black, a sample of 100 trajectories drawn from each model; cyan, the closest trajectory to the ground truth wILI from each sample of 100.

Fig 2 shows sample forecasts over wILI trajectories generated by each of these approaches and compares them to some alternatives described in S1 Appendix.

### Combining multiple methods: Stacking approach to model averaging

Forecasting systems that select effective combinations of predictions from multiple models can improve on the performance of the individual components, as demonstrated by their successful application in many domains. For each probability distribution and point prediction in a forecast, we treat the choice of an effective combination as a statistical estimation problem, and base each decision on the models’ behavior in leave-one-out cross-validation forecasts. Additional cross-validation analysis indicates that this approach achieves performance comparable to or better than the best individual component.

#### Background, motivation for combining forecasts

Methods that combine the output of different models, called “ensembles”, “multi-model ensembles”, “super-ensembles”, “model averages”, or various other names based on the domain and type of approach, have been applied successfully in many problem settings, improving upon the results of the best individual model. An ensemble approach is motivated in the context of seasonal epidemic forecasting by factors such as:

- **Model misspecification and overconfidence in distributional forecasts**: Many methods overlook the possibility of a significant proportion of observed outcomes, or assign otherwise inappropriate probabilities. These omissions and other mistakes are not identical across models; the gaps left by one component can be filled in by another.
- **Leveraging partially correlated errors in point predictions**: The point prediction errors of individual methods can vary in magnitude and are often only partially correlated with each other, allowing ensemble methods to improve performance, e.g., by highly weighting more accurate predictors, or by reducing the variance when combining multiple unbiased estimators.
- **Strengths and weaknesses in different targets**: Some methods may work well for certain forecasting targets, but have poor performance or fail to produce predictions for others; model averages can be smoothly adjusted to account for different behaviors for different targets.
- **Changes in performance within seasons**: Making predictions at the beginning, middle, and end of a season can be seen as different tasks, and the relative performance characteristics of the components may change based on the time of season (or whether it is around a holiday). Just as ensemble methods can account for distinct patterns based on forecasting target, they can be tailored to account for changes in behavior within a season.

We developed an adaptively weighted model average that consistently outperforms the best individual component. Other teams submitting forecasts to the FluSight comparison have concurrently developed other ensemble systems and found similar success [36, 37]. Our approach is distinguished from these other methods in that it very directly estimates the best model average weights for a given location, time, target, and evaluation metric.

#### A stacking approach to model averaging

For each location *l*, week *t*, target *i*, and evaluation metric *e*, we choose a (weighted) model average as the final prediction: an ensemble forecast of the form **Xw**, where

- **X** is the output of the *m* ensemble components—either (a) a row vector of point predictions with *m* entries, or (b) a matrix of distributional predictions with *m* columns—and
- **w** ∈ [0, 1]^*m*^ is a (column) vector of weights, one per component, with $\sum_{j=1}^{m}w_{j}=1$.

Variants of the same models, or methods based on related approaches or assumptions, may at times produce similar forecasts that commit the same errors while producing a misleading impression of consensus; a successful ensemble may need to consider not only the performance of each individual component, but also the relationships between the raw output of the components. To this end, we use a “stacking generalization” approach [34, 35], treating the selection of weights **w** for the current season, *S* + 1, as the task of frequentist estimation of the risk-optimal weight vector,

$$\begin{array}{c} w^{*}=\underset{\begin{array}{l} w\in {\left[0,1\right]}^{m}\\ \sum_{j=1}^{m}w_{j}=1 \end{array}}{\text{arg}\;\text{max}}E\left[\text{Score}\left(w,S+1,l,t,i,e\right)\right], \end{array}$$

based on leave-one-season-out cross-validation:

$$\hat{w}=\mu e_{\text{uniform}}+\left(1-\mu \right)\underset{\begin{array}{l} w\in {\left[0,1\right]}^{m}\\ \sum_{j=1}^{m}w_{j}=1 \end{array}}{\text{arg}\;\text{max}}\underset{\begin{array}{l} s^{\prime }\in \left\{1..S\right\},\\ l^{\prime },t^{\prime },i^{\prime },e^{\prime } \end{array}}{\sum }\begin{array}{c} \text{RelevanceWeight}\left(s^{\prime },l^{\prime },t^{\prime },i^{\prime },e^{\prime };S+1,l,t,i,e\right)\\ \cdot \text{CrossValidationScore}\left(w,s^{\prime },l^{\prime },t^{\prime },i^{\prime },e^{\prime }\right), \end{array}$$

where *μ* is an inflation factor that gives addition weight to the uniform component (**e**_uniform_ is a vector containing a 1 in the position corresponding to the uniform distribution component, and 0 in every other position). We changed the RelevanceWeight function used for real-time forecasts throughout the 2015/2016 season, but study only the following RelevanceWeight function in the cross-validation analysis of the adaptively weighted ensemble:

$$\begin{array}{c} \text{RelevanceWeight}\left(s,l,t,i,e;s^{\prime },l^{\prime },t^{\prime },i^{\prime },e^{\prime }\right)=\{\begin{array}{cc} 1, & |t-t^{\prime }|\leq 4,\;i=i^{\prime },\;e=e^{\prime }\\ 0, & \text{otherwise}. \end{array} \end{array}$$

A larger collection of cross-validation data can be considered by assigning relevance weights of 1 to additional training instances; relevance weights can also be gradually decreased for less similar data rather than jumping down to zero.

When *e* is the unibin or multibin log score:

- Using the rule of three [47] to estimate the frequency of events that we haven’t seen before, we chose $\mu =\frac{3}{S\cdot L}$ for most submissions. (Prior to the submission for 2015 EW43, we used a constant *μ* = 0.01 to guarantee a certain minimum log score.)
- The optimization problem is equivalent to fitting a mixture of distributions, and we can use the degenerate EM algorithm [48] to efficiently find the weights; convex optimization techniques such as the logarithmic barrier method are also appropriate.

When *e* is mean absolute error:

- We choose *μ* = 0 (and further, exclude the uniform distribution method from the ensemble entirely).
- This optimization problem is referred to as least absolute deviation regression or median regression, with linear inequality and equality constraints on the coefficients; we reformulate the problem as a linear program and use the lpSolve package [49] to find a solution.

We compare the “adaptive” weighting scheme above to two alternatives:

- **Fixed-weightset-based stacking**: the same approach as above, with the same *μ* selections but a different RelevanceWeight function:
  $$\begin{array}{c} \text{RelevanceWeight}\left(s,l,t,i,e;s^{\prime },l^{\prime },t^{\prime },i^{\prime },e^{\prime }\right)=\{\begin{array}{cc} 1, & e=e^{\prime }\\ 0, & \text{otherwise}; \end{array} \end{array}$$
  and
- **Uniform weights**: does not use the above stacking scheme; instead, for every prediction, assigns each component the same weight in the ensemble, $\frac{1}{m}$ (replacing $\hat{w}$ with $\frac{1}{m}\vec{1}$).

The ensemble and each of its components forecast the targets **Z**^*t*^ given a point or distributional estimate for *Y*_1..*T*_.

### Considering details of the data generation process

Two important features of ILINet data to consider in models and forecast evaluation are 1. timeliness and accuracy of initial wILI values for each week and subsequent updates to these values, and 2. changes in behavior on and around major holidays. We examine these details of the data generation process, describe how they are addressed in the delta density model, and demonstrate the importance of considering the update procedure when performing retrospective evaluation and prospective forecasting.

#### Modeling backfill updates to past wILI

When a wILI value is reported for a given week, it is not set in stone; as ILINet members provide “backfill” reports or revisions for past weeks and data is cleaned, wILI observations are updated accordingly. When generating a retrospective forecast, it is important to use the version of the data that would have been available at the time rather than the final revision in order to get a more appropriate estimate of the future performance of a forecasting system. Furthermore, forecasting performance can be improved by modeling and “backcasting” these backfill updates, accounting for the following sources of error:

- **Biased early reports**: earlier wILI versions are generally biased downwards early in the in-season, and upwards towards the end of the in-season, which may lead to forecasts of lower, later peaks early in the season, and of longer epidemic duration later in the season;
- **Overconfident short-term distributional forecasts**: since updates in wILI can cause “observed” data, e.g., of the wILI at the presumed peak week, to shift, ignoring backfill may lead to “thin”, overconfident forecast distributions;
- **Revisions of “observed” seasonal targets**: wILI updates sometimes cause large changes in the apparent onset week or peak week when there are bumps or multiple peaks in the trajectory: wILI updates can cause a measurement to change from above the CDC baseline to below (or vice versa), or for an earlier, lower peak to rise above a later peak (or vice versa); ignoring backfill updates can cause models to completely miss some possibilities when these targets appear to be determined. A similar type of error can arise from revisions to the peak height value (regardless of whether the peak week changes); even small updates can result in large unibin log score penalties.

We estimate the distribution of backfill updates using the residual density method with *t*_1_ = 0, *t*_2_ = *t*, $X_{1..t}^{}=W_{1..t}^{t}$ the latest version of wILI available, *Y*_1..*t*_ the corresponding final revisions, and **R**^*u*^ = [*Y*_*u*−1_]. The weight given to a historical nonfinal-to-final residual is based on three factors:

- **Lag amount**: later revisions of wILI values tend to be closer to the final revision than earlier revisions are; thus, when estimating the distribution of *n*-week-old wILI to finalized wILI residuals, only *n*-week-old wILI to finalized wILI data is considered; backfill data for other lags is ignored (i.e., has zero weight);
- **The current season’s nonfinal wILI value**: historical backfill updates with nonfinal wILI values closer to the nonfinal wILI value from the current season are given greater weights according to a Gaussian kernel (with bandwidth based on a rule for kernel density estimation of the historical nonfinal wILI values);
- **Epi week of observation**: since the backfill pattern changes throughout a season, historical backfill updates corresponding to nearby epi weeks are weighted more highly than those from a different time of the season, using a Laplacian kernel (with an arbitrarily selected bandwidth).

The bandwidth of the density estimate is based on a kernel density estimate of the nonfinal-to-final residuals.

The backcasting method is modular and can combine with any forecaster expecting ground truth wILI as input. The straightforward approach is to sample a few thousand trajectories from the backfill simulator, feed each of these into the forecaster to obtain a trajectory or a distribution over targets, and aggregate the results. Some forecasting methods in the Delphi-Stat ensemble do not have a simple way to quickly generate single-trajectory forecasts, so we also use alternative approaches to reduce computation, such as randomly pairing backcasts and trajectory forecasts, where the trajectory forecasts are efficiently generated in batch, based on the pointwise mean of the backcasts.

#### Latency of initial wILI value and “nowcasting”

The initial ILINet wILI value for a given “target” week (from Sunday to Saturday) is typically released on Friday of the following week. Data sources with lower latency and higher temporal resolution can be used to prepare wILI estimates (“nowcasts”) earlier in the following week or even during the target week itself. More generally, auxiliary data for past and current weeks can improve not only models of disease activity in these weeks but also forecasts of future disease activity. Given a backcaster than simulates finalized data for past weeks *Y*_1..*t*_ given observed ILINet and auxiliary data, a nowcaster that simulates *Y*_*t*+1_ given these observations and (a simulated) *Y*_1..*t*_, and a forecaster that simulates *Y*_*t*+2..*T*_ given these observations and (a simulated) *Y*_1..*t*+1_, we can sample from an enhanced model of *Y*_1..*T*_ (given the latest wILI observations $W_{1..t}^{t}$, previous versions of wILI, and auxiliary data) using the following procedure:

1. Repeatedly draw a random value $Y_{1..t}^{\text{sim}}$ for *Y*_1..*T*_ by:
  1. drawing a random value $Y_{1..t}^{\text{sim}}$ for *Y*_1..*t*_ conditioned on the observed data, using the backcaster, then
  2. drawing a random value $Y_{t+1}^{\text{sim}}$ for *Y*_*t*+1_ conditioned on the observed data and $Y_{1..t}^{}=y_{1..t}^{\text{sim}}$, using the nowcaster, then
  3. drawing a random value $Y_{t+2..T}^{\text{sim}}$ for *Y*_*t*+2..*T*_ conditioned on the observed data and $Y_{1..t+1}^{}=Y_{1..t+1}^{\text{sim}}$, using the forecaster, then
  4. combine $Y_{1..t}^{\text{sim}}$, $Y_{t+1}^{\text{sim}}$, and $Y_{t+2..T}^{\text{sim}}$ into a single (random) trajectory $Y_{1..T}^{\text{sim}}$, and
2. Collect these individual, randomly drawn trajectories into a list (i.e., a random sample).

As with the earlier method of combining backcasts and forecasts without a nowcaster, this procedure may be too computationally expensive for some implementations of some forecasters; we use these steps exactly with the delta density methods, for example, but consider modifications and approximations for some other forecasters.

This methodology can be applied in conjunction with one of many available nowcasters. We focus on ILI-Nearby [50, 51], which produces nowcasts for wILI by fusing together several “sensors” using another type of stacked generalization, where each sensor is also a nowcast of wILI data; we reproduce a list of references from [50] on other methodologies for nowcasting and incorporating auxiliary data here [15, 28, 31, 52–71] along with some more recent work [27, 29, 30], with special note of other work using multiple auxiliary data sources [29] or nowcasters [71]. We consider four distributional nowcasters:

- $Y_{t+1}^{\text{sim}}$ produced by the forecaster, i.e., not using separate nowcasts at all—the basis for all performance estimates unless otherwise noted, as no nowcasts were incorporated into the Delphi-Stat forecasts for the 2015/2016 season;
- $Y_{t+1}^{\text{sim}}$ following a normal distribution with mean and standard deviation given by the ILI-Nearby nowcasting system (ignoring the backcaster’s output);
- $Y_{t+1}^{\text{sim}}$ following a Student’s *t* distribution with two degrees of freedom, centrality parameter set to the ILI-Nearby point estimate, and scale parameter set to the ILI-Nearby standard deviation estimate, intended to be a wide-tailed variant of the above (ignoring the backcaster’s output);
- an ensemble of first and third approaches, with associated weights (probabilities) of 15% and 85% respectively. (The choice of weights was inherited from a similar approach that mixed “1 wk ahead” delta density forecasts with nowcasts, rather than ensemble forecasts (including a uniform component) based on these two approaches; a nowcast weight of 85% was selected on a limited amount of out-of-sample (preseason) forecasts to maximize log score.)

#### Holiday effects

**Fig 3:**
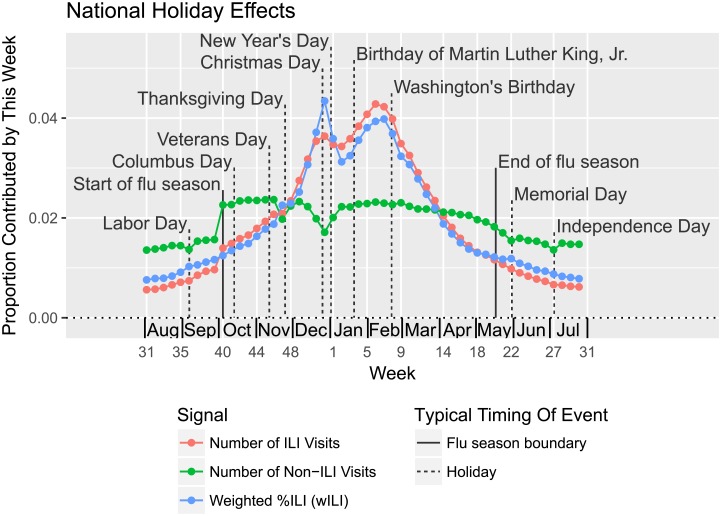
On average, wILI is higher on holidays than expected based on neighboring weeks. Weekly trends in wILI values, as expressed by the contribution of a each week to a sum of wILI values from seasons 2003/2004 to 2015/2016, excluding 2008/2009 and 2009/2010 (which include portions of the 2009 influenza pandemic), show spikes and bumps upward on and around major holidays. (U.S. federal holidays are indicated with event lines.) The number of non-ILI visits to ILINet health care providers spikes downwards on holidays (disproportionately with any drops in the number of ILI visits), contributing to higher wILI. The number of ILI visits generally declines in the second half of the winter holiday season, causing winter holiday peaks to appear even higher relative to nearby weeks. In addition to holiday effects, we see that average ILINet participation jumps upward on epi week 40, and gradually tapers off later in the season and in the off-season.

On average, wILI tends to be higher on holidays and during the winter holiday season than would be expected based on interpolating observations from nearby weeks [21, 50], as seen in Fig 3. Sharp rises and drops in wILI are common from early or mid-December to early January (roughly coinciding with a four week period beginning with epi week 50), with either the season’s peak or a lower, secondary peak commonly occurring on epi week 52. This pattern appears to arise from two factors:

- spikes downward in the number of non-ILI visits during the holiday season (corresponding to increases in wILI), perhaps caused by patients choosing not to visit the doctor for less serious issues on holidays, and
- decreases in the average number of ILI visits at the end of the holidays, perhaps due to decreased transmission of ILI during holidays, which make the preceding increases in wILI appear even sharper.

Similarly, there are spikes or minor blips downward in the average number of non-ILI visits (which can result in small increases in wILI) associated with Thanksgiving Day; Labor Day; Independence Day; Memorial Day; Birthday of Martin Luther King, Jr.; Washington’s Birthday; Columbus Day; and perhaps other holidays. The spike upward in wILI at Thanksgiving can push wILI unexpectedly over the onset threshold, and holiday effects may help explain the surprising frequency at which peaks occur on epi week 7 but not neighboring weeks. Additional age-specific patterns may be obscured by this analysis of aggregate ILI and non-ILI visit counts.

#### Impact of holiday effects on choice of kernels

Each holiday above occurs at roughly the same time of year every year, falling on one of two possible epi weeks. Thus, models that predict behavior at a given epi week by prioritizing or focusing solely on past behavior at that given epi week will automatically perform a rough adjustment for holiday effects. This factor informs our decision to use historical data only from corresponding weeks in the Markovian delta density method, and a truncated Laplacian kernel with narrower width near winter holidays in the extended delta density method. Specifically, for the extended delta density method, we choose the half-width of the kernel to be *l*^*u*^ = min{10, max{0, |*u* − 22| − 1}}, which assigns *l*^*u*^ = 0 for *u* within one week of epi week 52, and larger *l*^*u*^’s the farther *u* is from this time period, up to a maximum value of 10.

## Results

### 2015/2016 FluSight comparison

During the 2015/2016 FluSight comparison, we submitted weekly, prospective forecasts from three forecasting systems:

- **Delphi-Stat**: an adaptively weighted ensemble of instance-based statistical forecasting methods, and the topic of this paper;
- **Delphi-Archefilter**: forms an empirical (rather than mechanistic) process model describing wILI trajectories, and incorporates both wILI and multiple forms of digital surveillance data using statistical filtering techniques [50]; and
- **Delphi-Epicast**: wisdom-of-crowds approach based on combining predictions submitted by several human participants [72].

**Fig 4:**
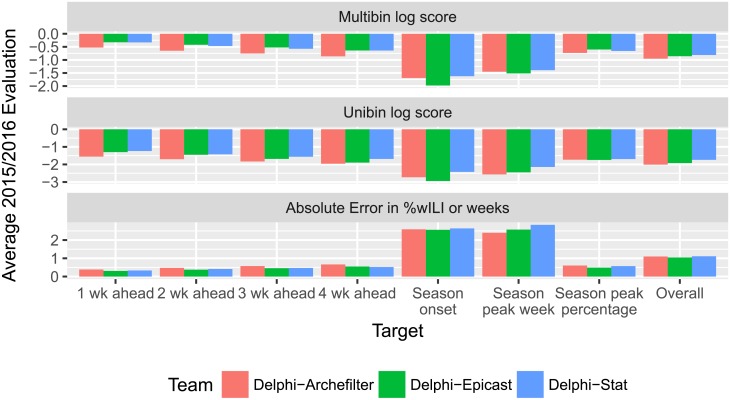
The three Delphi systems had similar overall scores; Delphi-Stat gave the best distributional forecasts overall, while Delphi-Epicast gave the best point predictions overall. These bar plots contain evaluations for the 2015/2016 season, averaged across 11 locations and 29 forecast weeks, for each target and evaluation metrics. Shorter bars indicate better performance. Each entry for a specific target is an average of 319 evaluations, giving a total of 2233 evaluations overall for each system. This figure’s data is shown in tabular form in S2 Appendix.

Our past and ongoing forecasts, as well as Python [73] and R [46] code for components of the systems used to generate them, are publicly available online [74–76]. Changes made to Delphi-Stat throughout the 2015/2016 season are described in S5 Appendix. These three forecasting systems were ranked as the top three in the 2015/2016 comparison in terms of overall multibin score, with Delphi-Stat at the top. Fig 4 shows the performance of the three Delphi forecasting systems, broken down by evaluation metric and forecasting target. S1 and S2 Figs. show the multibin scores broken down by location and by forecasting week. Delphi-Stat had consistently strong aggregate multibin scores across different targets, locations, and forecasting weeks, and the best overall multibin log score of all FluSight 2015/2016 submissions. Delphi-Stat’s unibin log score evaluations relative to the other two Delphi systems seem similar to or better than the corresponding multibin log score evaluations, as Delphi-Stat has the best unibin score of the three for each target rather than just overall; this observation seems natural since Delphi-Stat was developed to optimize unibin log score, and may suggest that optimizing for multibin log score rather than unibin log score when selecting ensemble weights or as a post-processing step could produce multibin log score improvements. However, the system’s point predictions, while optimized for the mean absolute error metric, were less accurate than (but still competitive with) the other two when averaged across all predictions.

### Cross-validation analysis

Prospective forecast evaluation ensures that performance estimates are truly out-of-sample, not inflated by design decisions or model fits that are influenced by the evaluation data; however, such evaluation data is not readily generated, as it is expensive in terms of physical time: new wILI observations arrive once per week, and performance can vary significantly from season to season and from week to week. The evaluations from the 2015/2016 comparison may be noisy due to these season-to-season fluctuations. To address this issue, we use pseudo-out-of-sample retrospective analysis to provide more stable estimates of performance. Specifically, we use leave-one-season-out cross-validation: for each evaluation season *s*, we form and evaluate retrospective forecasts for *s* at every evaluation week using all training seasons except for *s* as inputs to the forecasting methods as if they were past seasons. (We exclude seasons prior to 2010/2011 from the evaluation set because records of HHS region ILINet data revisions are only available beginning in late 2009. We exclude seasons prior to 2003/2004 from the training set because year-round ILINet observations, which are required by some of the ensemble components, started in 2003. The 2009/2010 season—containing the peak of the 2009 pandemic according to our adjusted definition of “season”—is also removed from the training set. Finally, we do not include the season currently underway (*S* + 1) in evaluation or training as it has not been completely observed.) Using cross-validation prevents most direct model fitting to evaluation data, and basing design decisions on motivations other than the effects on cross-validation evaluation helps limit fitting through iterative design.

**Fig 5:**
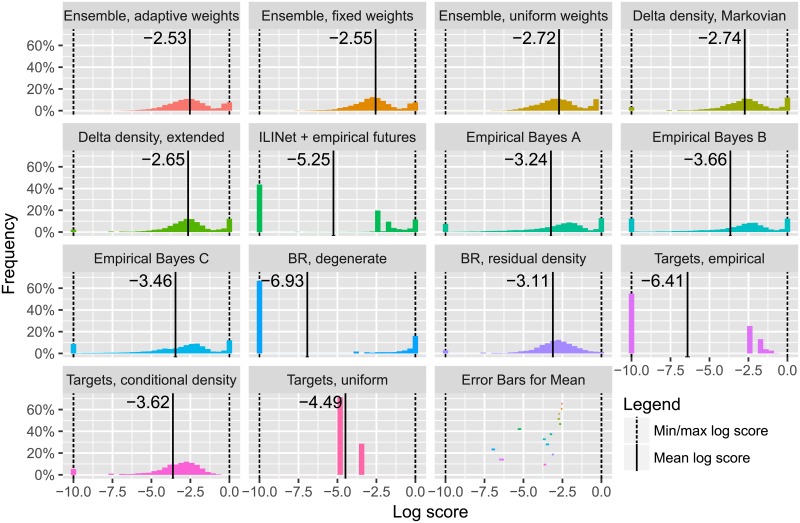
Delta and residual density methods cover more observed events and attain higher average log scores than alternatives operating on seasons as a unit; ensemble approaches can eliminate missed possibilities while retaining high confidence when justified. This figure contains histograms of cross-validation log scores for a variety of forecasting methods, averaged across seasons 2010/2011 to 2015/2016, all locations, forecast weeks 40 to 20, and all forecasting targets. A solid black vertical line indicates the mean of the scores in each histogram, which we use as the primary figure of merit when comparing forecasting methods; a rough error bar for each of these mean scores is shown as a colored horizontal bar in the last panel, and as a black horizontal line at the bottom of the corresponding histogram if the error bar is wider than the thickness of the black vertical line.

Fig 5 shows the distribution of log scores for several forecasting methods, described earlier in the text and in S1 Appendix, and the three ensemble approaches specified earlier in the text. Except for the uniform distribution and ensembles, all forecasting methods miss some possibilities completely, reporting unreasonable probabilities less than exp(−10) ≈ 0.0000454 for events that actually occurred. In these situations, the log score has been increased to the cap of −10 (as CDC does for multibin log scores). Delta and residual density forecasting methods (Delta density, Markovian; Delta density, extended; and BR, residual density) are less likely to commit these errors than other non-ensemble, non-uniform approaches, and have higher average log scores. Ensemble approaches combine forecasts of multiple components, missing fewer possibilities, and ensuring that a reasonable log score is obtained by incorporating the uniform distribution as a component. For the full Delphi-Stat ensemble, the main advantage of the ensemble over its best component appears to be successfully filling in possibilities missed by the best component with other models to avoid -10 and other low log scores appears, while for ensembles of subsets of the forecasting methods, there are other benefits; S3 Appendix shows the impact of these missed possibilities and the log score cap.

Fig 5 also includes estimates of the mean log score for each method and rough error bars for these estimates. We expect there to be strong statistical dependence across evaluations for the same season and location, and weaker dependencies between different seasons and locations; thus, the most common approaches to calculating standard errors, confidence intervals, and hypothesis test results will be inappropriate. Properly accounting for such dependencies and calibrating intervals and tests is an important but difficult task and is left for future investigation. We use “rough standard error bars” on estimates of mean evaluations: first, the relevant data (e.g., all cross-validation evaluations for a particular method and evaluation metric) is summarized into one value for each season-location pair by taking the mean of all evaluations for that season-location pair; we then calculate the mean and standard error of the mean of these season-location values using standard calculations as if these values were independent. Under some additional assumptions which posit the existence of a single underlying true mean log score for each method, these individual error bars—or rough error bars for the mean difference in log scores between pairs of ethods—suggest that the observed data is unlikely to have been recorded if the true mean log score of the extended delta density method were greater than that of the adaptively weighted ensemble, or if the true mean log score of the “Empirical Bayes A” method were greater than the extended delta density method. The mean and rough standard error estimates in Fig 5 also appear in tabular form in S4 Appendix.

Methods that model wILI trajectories and “pin” past wILI to its observed values have a large number of log scores near 0 because they are often able to confidently “forecast” many onsets and peaks that have already occurred; ensemble methods also have a large number of log scores near 0. Note that these scores are closer to 0 for ensembles that optimize weighting of different methods than for the ensemble with uniform weights. For this particular set of forecasting methods, targets, and evaluation seasons:

- the uniformly weighted ensemble has lower average log score than the best individual component (extended delta density),
- using the stacking approach to assign weights to ensemble components improves ensemble performance significantly and gives higher average log score than the best individual component,
- the adaptive weighting scheme does not provide a major benefit over a fixed-weight scheme using a single set of weights for each evaluation metric.

When given subsets of these forecasting methods as input, with regard to average performance:

- the uniformly weighted ensemble often outperforms the best individual, but is sometimes slightly (≈ 0.1 log score) worse;
- the stacking approach improves upon the performance of the uniformly weighted ensemble; and
- the adaptive weighting scheme’s performance is equal to or better than that of the fixed-weight scheme, sometimes improving on the log score by ≈ 0.1. The adaptive weighting scheme’s relative performance appears to improve with more input seasons, fewer ensemble components, and increased variety in underlying methodologies and component performance. These trends suggest that using wider RelevanceWeight kernels, regularizing the component weights, or considering additional data from 2003/2004 to 2009/2010, for which ground truth wILI but not weekly ILINet reports are available, may improve the performance of the adaptive weighting scheme. In addition to these avenues for possible improvement in ensemble weights for the components presented in Fig 5, the adaptive weighting scheme provides a natural way of incorporating forecasting methods that generate predictions for only a subset of all targets, forecast weeks, or forecast types (distributional forecast or point prediction). For example, in the 2015/2016 season, we incorporated a generalized additive model that provided point predictions (and later, distributional forecasts) for peak week and peak height given at least three weeks of observations from the current season.

**Fig 6:**
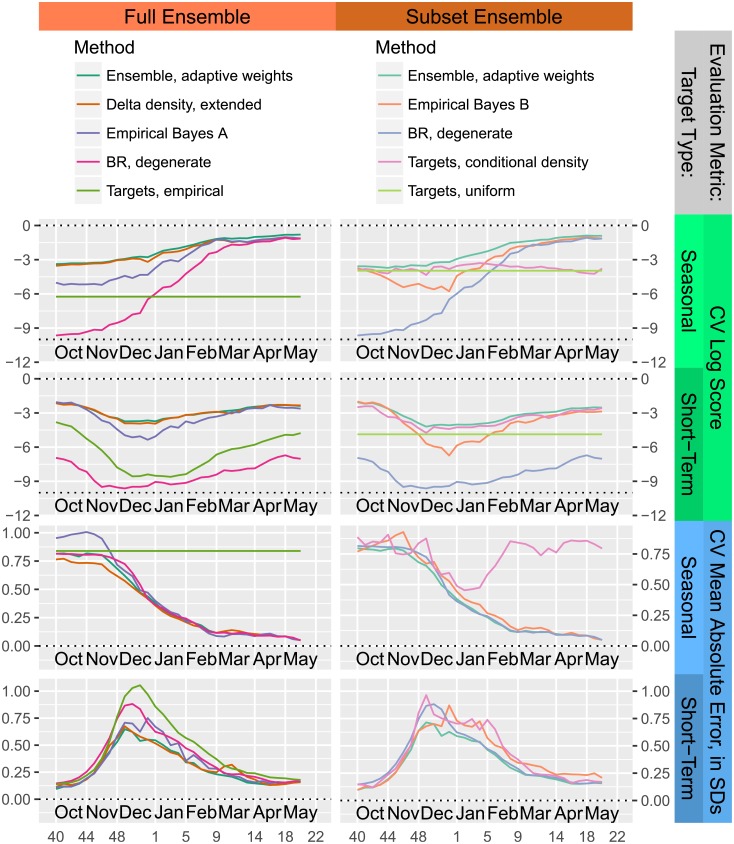
The ensemble method matches or beats the best component overall, consistently improves log score across all times, and, for some sets of components, can provide significant improvements in both log score and mean absolute error. These plots display cross-validation performance for two ensembles and some components broken down by evaluation metric, target type, and forecast week; each point is an average of cross-validation evaluations for all 11 locations, seasons 2010/2011 to 2015/2016, and all targets of the given target type; data from the appropriate ILINet reports is used as input for the left-out seasons, while finalized wILI is used for the training seasons. Top half: log score evaluations (higher is better); bottom half: mean absolute error, normalized by the standard deviation of each target (lower is better). Left side: full Delphi-Stat ensemble, which includes additional methods not listed in thelegend; right side: ensemble of the three methods listed in the legend, plus a uniform distribution component for distributional forecasts. Many components of the full ensemble are not displayed. The “Targets, uniform” method is excluded from any mean absolute error plots as it was not incorporated into the point prediction ensembles.

Fig 6 shows a subset of the cross-validation data used to form the ensemble and evaluate the effectiveness of the ensemble method, for two sets of components: one using all the components of Delphi-Stat, and the other incorporating three of the lower-performance components and a uniform distribution for distributional forecasts. The Delphi-Stat ensemble near-uniformly dominates the best component, extended delta density, in terms of log score, and has comparable mean absolute error overall. The ensemble approach produces greater gains for the smaller subset of methods, surpassing not only its best components, but all forecasting methods in the wider Delphi-Stat ensemble except for the delta density approaches.

**Fig 7:**
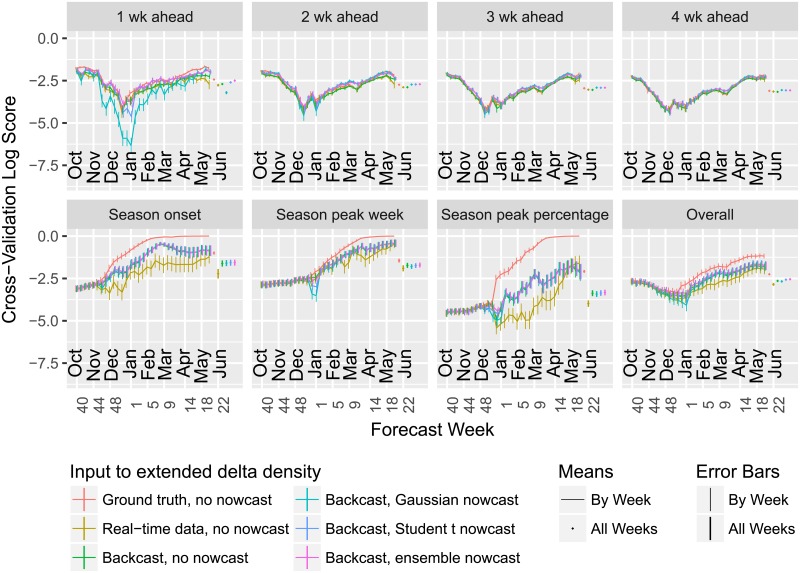
Using finalized data for evaluation leads to optimistic estimates of performance, particularly for seasonal targets, “backcasting” improves predictions for seasonal targets, and nowcasting can improve predictions for short-term targets. Mean log score of the extended delta density method, averaged across seasons 2010/2011 to 2015/2016, all locations, all targets, and forecast weeks 40 to 20, both broken down by target and averaged across all targets (“Overall”). Rough standard error bars for the mean score for each target (or overall) appear on the right, in addition to the error bars at each epi week.

Fig 7 shows cross-validation performance estimates for the extended delta density method based on three evaluation schemes:

- **Ground truth, no nowcast**: the ground truth wILI for the left-out season up to the forecast week is provided as input, resulting in an optimistic performance estimate;
- **Real-time data, no nowcast**: the appropriate wILI report is used for data from the left-out season, but no adjustment is made for possible updates; this performance estimate is valid, but we can improve upon the underlying method;
- **Backcast, no nowcast**: the appropriate wILI report is used for data from the left-out season, but we use a residual density method to “backcast” updates to this report; this performance estimate is valid, and the backcasting procedure significantly improves the log score;
- **Backcast, Gaussian nowcast**: same as “Backcast, no nowcast” but with another week of simulated data added to the forecast, based on a Gaussian-distributed nowcast; and
- **Backcast, Student *t* nowcast**: same as “Backcast, Gaussian nowcast” but using a Student *t*-distributed nowcast in place of the Gaussian nowcast.
- **Backcast, ensemble nowcast**: same as the previous two but using the ensemble nowcast (which combines “no nowcast” with “Student *t* nowcast”).

For every combination of target and forecast week, using ground truth as input rather than the appropriate version of these wILI observations produces either comparable or inflated performance estimates.

Using the “backcasting” method to model the difference between the ground truth and the available report helps close the gap between the update-ignorant method. The magnitude of the performance differences depends on the target and forecast week. Differences in mean scores for the short-term targets are small and may be reasonably explained by random chance alone; the largest potential difference appears to be an improvement in the “1 wk ahead” target by using backcasting. More significant differences appear in each of the seasonal targets following typical times for the corresponding onset or peak events; most of the improvement can be attributed to preventing the method from assigning inappropriately high probabilities (often 1) to events that look like they must or almost certainly will occur based on available wILI observations for past weeks, but which are ultimately not observed due to revisions of these observations. The magnitude of the mean log score improvement depends in part on the resolution of the log score bins; for example, wider bins for “Season peak percentage” may reduce the improvement in mean log score (but would also shrink the scale of all mean log scores). Similarly, the differences in scores may be reduced but not eliminated by use of multibin scores for evaluation or ensembles incorporating uniform components for forecasting.

Using the heavy-tailed Student *t* nowcasts or nowcast ensemble appears to improve on short-term forecasts without damaging performance on seasonal targets. The performance of the nowcast ensemble is further explored in S5, S6, S7, S8, S9, S10, S11 and S12 Figs. The Gaussian nowcast has a similar effect as the other nowcasters except on the “1 wk ahead” target that it directly predicts: its distribution is too thin-tailed, resulting in lower mean log scores than using the forecaster by itself on this target.

## Discussion

Delphi-Stat forecasts submitted to the 2015/2016 comparison were based solely on wILI observations from the 2015/2016 “pre-season” (EW21–EW39) and season (EW40–EW20) and nonmechanistic models (with a majority of the ensemble weight assigned to the delta and residual density based methods). Additional data and mechanistic models dealing with categories of ILI or type and subtype of influenza, climate, digital surveillance, season-to-season patterns, spatial interaction, etc. were not incorporated. We do think that these types of data are useful, but analyzing their dynamics and effects on wILI is complicated by the fact that the smallest geographical units for which real-time wILI data is readily available for the entire US are HHS regions (on a weekly time scale). We expect that mechanistic components incorporating climate data and separating diseases, types, and subtypes will be more useful when we are able to model, forecast, and validate data at a finer geographical resolution, ideally at the metro area level. Similarly, we believe that digital surveillance data is useful; in fact, we currently use a sensor fusion framework to combine several such data sources and short-term forecasters to produce “nowcasts” for the current week [50, 51], and improve the performance of forecasting methods by incorporating these nowcasts in a manner similar to the ILINet-based backcasts.

During development and throughout this manuscript, we have focused on (thresholded) unibin log score as a (near-)proper, simple-to-implement metric for distributional forecasts. CDC FluSight organizers, on the other hand, selected exponentiated mean thresholded multibin log scores over the entire influenza season as the evaluation metric for forecast comparisons to 1. encourage high-quality distributional predictions rather than point predictions, for better understanding of the risk of certain scenarios, 2. make the scoring metric more accessible to policymakers than unibin and non-exponentiated variants, and 3. avoid −∞ scores due to a single forecaster mistake or unmodeled data revisions. We believe that it is up to policymakers to decide whether these forecasts are ready for use in decision support at the current level of accuracy. For other potential users and forecast comparisons, we provide absolute error evaluations for all targets in 2015/2016 in Fig 4 and S2 Appendix, as well as absolute error and percent absolute error for short-term targets from cross-validation in S5, S6, S7, S8, S9, S10, S11 and S12 Figs.

### Conclusion

The delta density forecasting method, stacking-based adaptively weighted ensemble, distributional “backcasts” of wILI updates, and nowcasts from ILI-Nearby provide significant improvements upon other individual forecasting approaches that we considered. Promising avenues for further improvements include refining the methodology to rely less on arbitrary and heuristic feature, kernel, bandwidth, and parameter selections, regularization of ensemble weights, incorporating conditional density estimators from statistical literature, and using additional data sources and finer-resolution data models.

## Supporting information

S1 Fig Multibin log scores for the 2015/2016 season—Averaged across 7 targets and 29 forecast weeks, broken down by location. Smaller bars indicate better performance. Each bar is an average of 203 evaluations. This figure’s data is included in tabular form in S2 Appendix.
(PDF)

S2 Fig Multibin log scores for the 2015/2016 season—Averaged across 7 targets and 11 locations, broken down by forecast week. Higher log scores indicated better performance. Each point is an average of 77 evaluations. This figure’s data is included in tabular form in S2 Appendix.
(PDF)

S3 Fig Unibin log score of the delta density method with backcasting—Averaged across targets and locations, broken down by season and epi week. (PDF)

S4 Fig Unibin log score of the delta density method with backcasting—Averaged across targets and seasons, broken down by location and epi week. (PDF)

S5 Fig Mean absolute error of short-term wILI point predictions of the extended delta density method with backcasting and ensemble nowcasting, and of wILI reports—Averaged across seasons, locations, and epi weeks, broken down by timeliness of the estimates. (PDF)

S6 Fig Empirical cumulative distribution function of the absolute error of short-term wILI point predictions of the extended delta density method with backcasting and ensemble nowcasting, and of wILI reports—Combined across seasons, locations, and epi weeks, broken down by timeliness of the estimates. (PDF)

S7 Fig Mean absolute error of short-term wILI point predictions of the extended delta density method with backcasting and ensemble nowcasting, and of wILI reports—Averaged across seasons and epi weeks, broken down by location and timeliness of the estimates. (PDF)

S8 Fig Empirical cumulative distribution function of the absolute error of short-term wILI point predictions of the extended delta density method with backcasting and ensemble nowcasting, and of wILI reports—Combined across seasons and epi weeks, broken down by location and timeliness of the estimates. (PDF)

S9 Fig Mean absolute percent error of short-term wILI point predictions of the extended delta density method with backcasting and ensemble nowcasting, and of wILI reports—Averaged across seasons, locations, and epi weeks, broken down by timeliness of the estimates. (PDF)

S10 Fig Empirical cumulative distribution function of the absolute percent error of short-term wILI point predictions of the extended delta density method with backcasting and ensemble nowcasting, and of wILI reports—Combined across seasons, locations, and epi weeks, broken down by timeliness of the estimates. (PDF)

S11 Fig Mean absolute percent error of short-term wILI point predictions of the extended delta density method with backcasting and ensemble nowcasting, and of wILI reports—Averaged across seasons and epi weeks, broken down by location and timeliness of the estimates. (PDF)

S12 Fig Empirical cumulative distribution function of the absolute percent error of short-term wILI point predictions of the extended delta density method with backcasting and ensemble nowcasting, and of wILI reports—Combined across seasons and epi weeks, broken down by location and timeliness of the estimates. (PDF)

S1 Appendix Description of all ensemble components in the 2015/2016 Delphi-Stat forecasting system. (PDF)

S2 Appendix Tabular evaluations of 2015/2016 forecasts. (PDF)

S3 Appendix “Missed possibilities” and -10 log score threshold. (PDF)

S4 Appendix Tabular evaluations of CV forecasts. (PDF)

S5 Appendix Log of changes to Delphi-Stat throughout the 2015/2016 season and for cross-validation analysis. (PDF)
