# Supplementary figures and images for: Nonmechanistic forecasts of seasonal influenza with iterative one-week-ahead distributions
Source: PLoS Comput Biol. 2018 Jun 15;14(6):e1006134. doi: 10.1371/journal.pcbi.1006134 (PMC6034894; doi:10.1371/journal.pcbi.1006134)

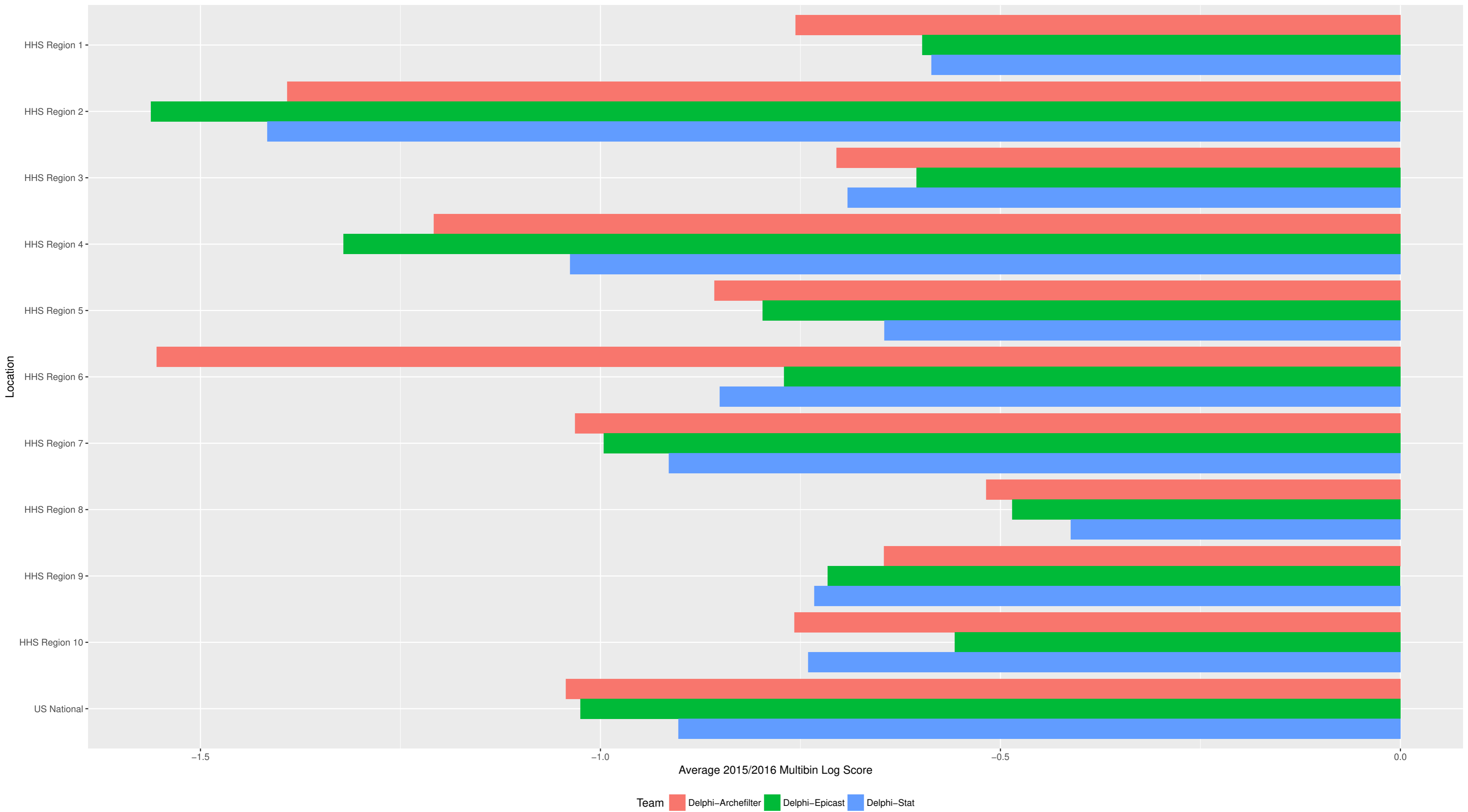

Supplement: S1 Fig — Smaller bars indicate better performance. Each bar is an average of 203 evaluations. This figure’s data is included in tabular form in S2 Appendix. (PDF) [file pcbi.1006134.s001.pdf]

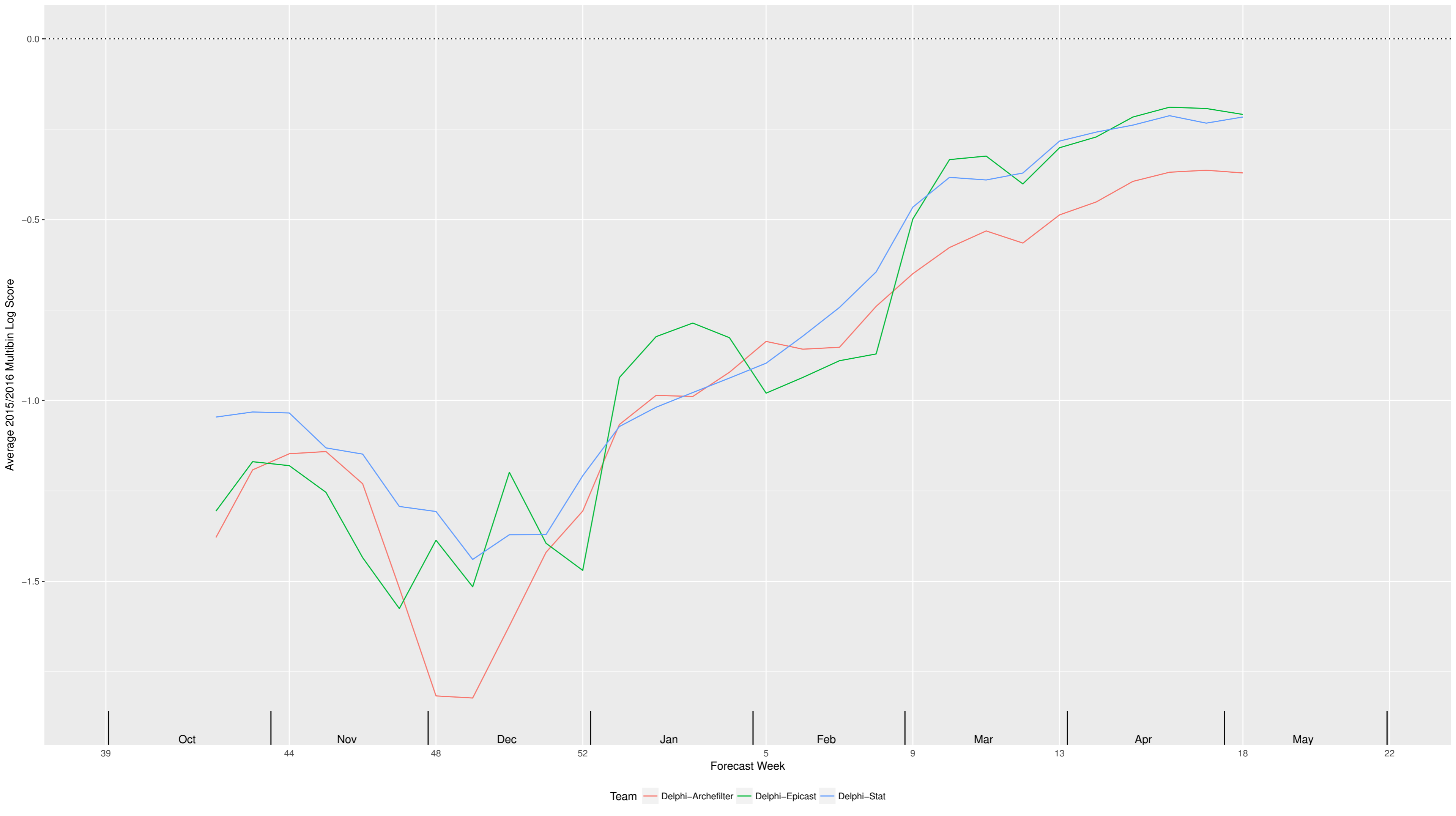

Supplement: S2 Fig — Higher log scores indicated better performance. Each point is an average of 77 evaluations. This figure’s data is included in tabular form in S2 Appendix. (PDF) [file pcbi.1006134.s002.pdf]

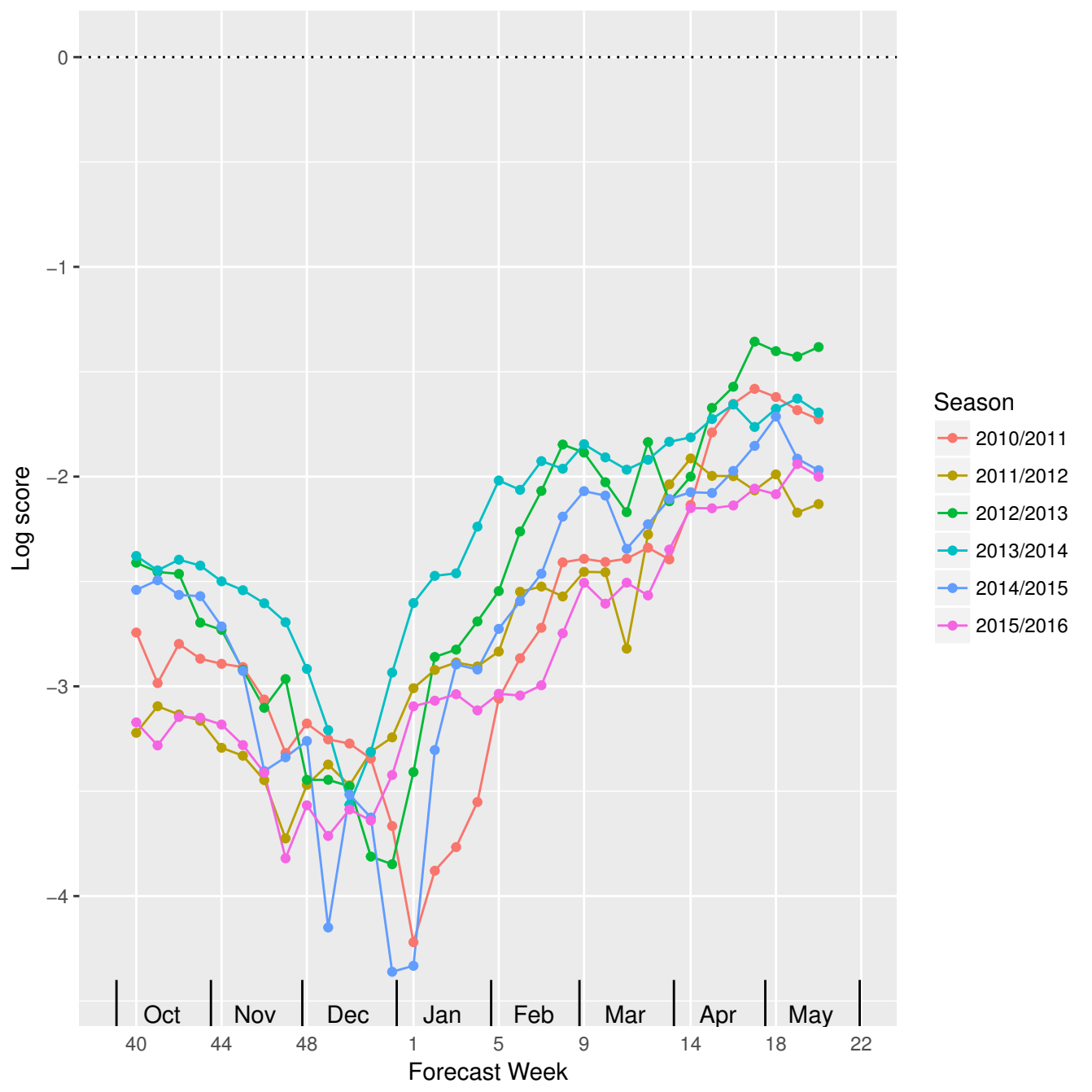

Supplement: S3 Fig — (PDF) [file pcbi.1006134.s003.pdf]

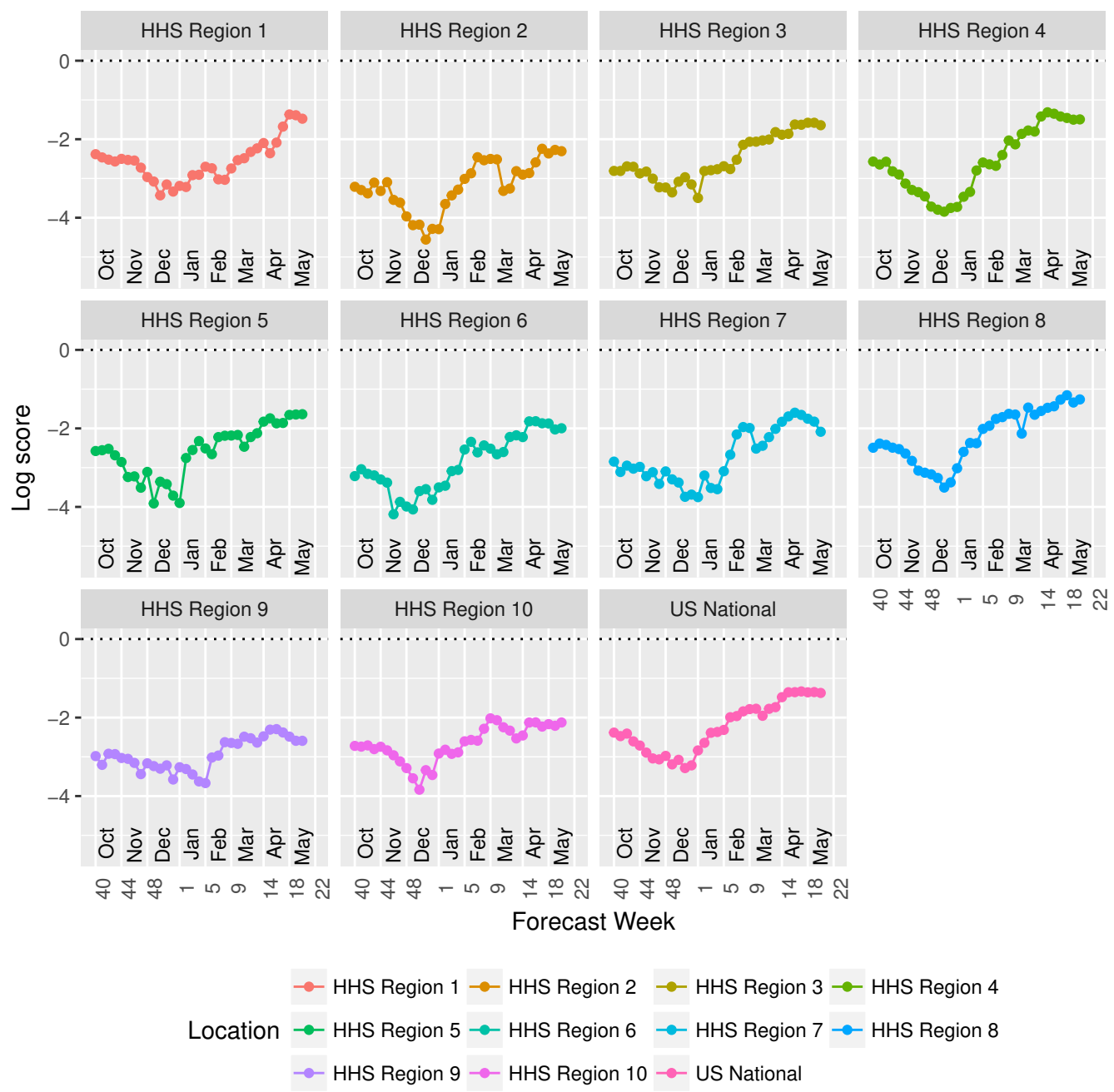

Supplement: S4 Fig — (PDF) [file pcbi.1006134.s004.pdf]

# Absolute Error in wILI Estimates, Averaged across 10 HHS Regions + National

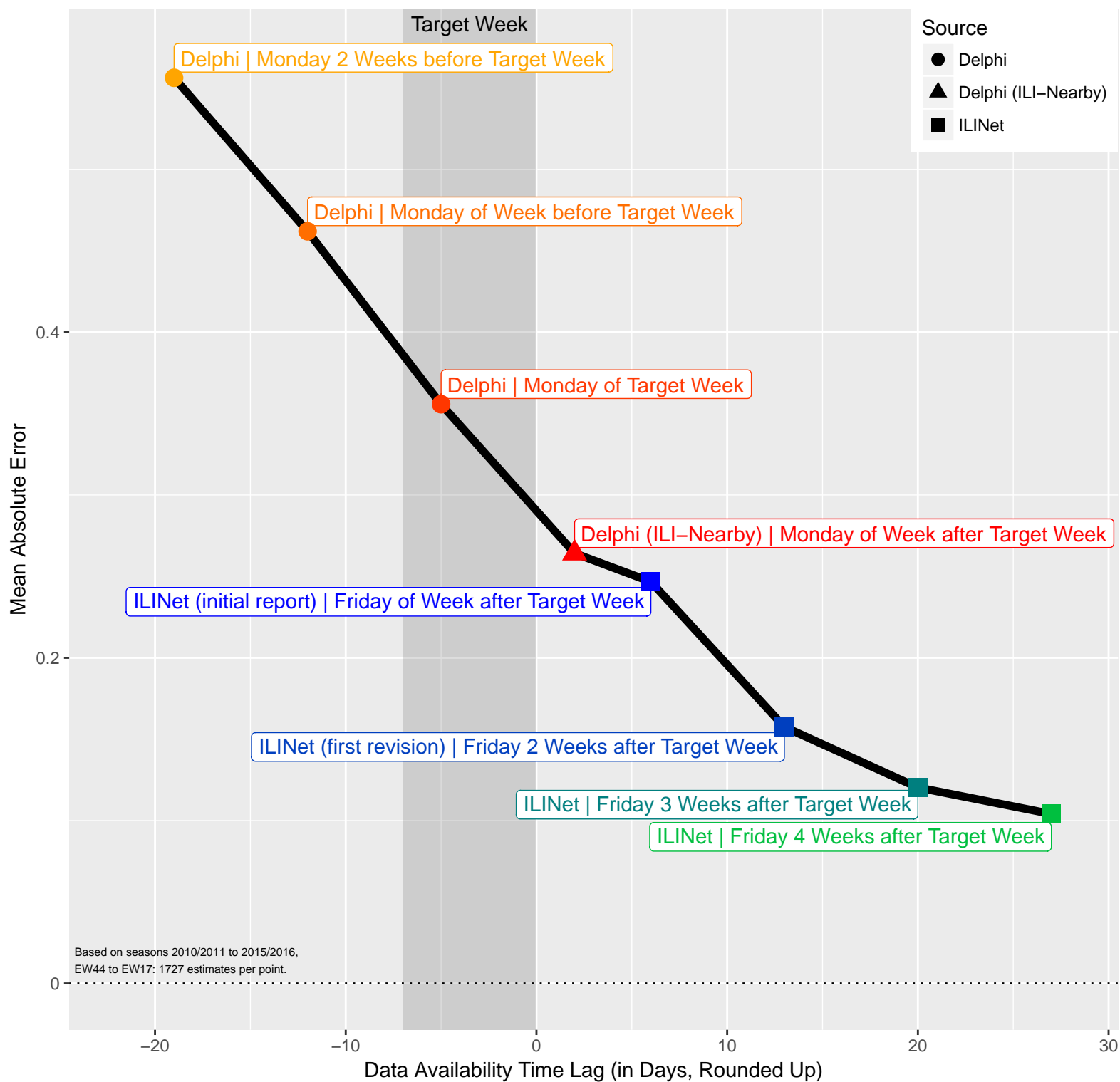

Supplement: S5 Fig — (PDF) [file pcbi.1006134.s005.pdf]

# Absolute Error in wILI Estimates

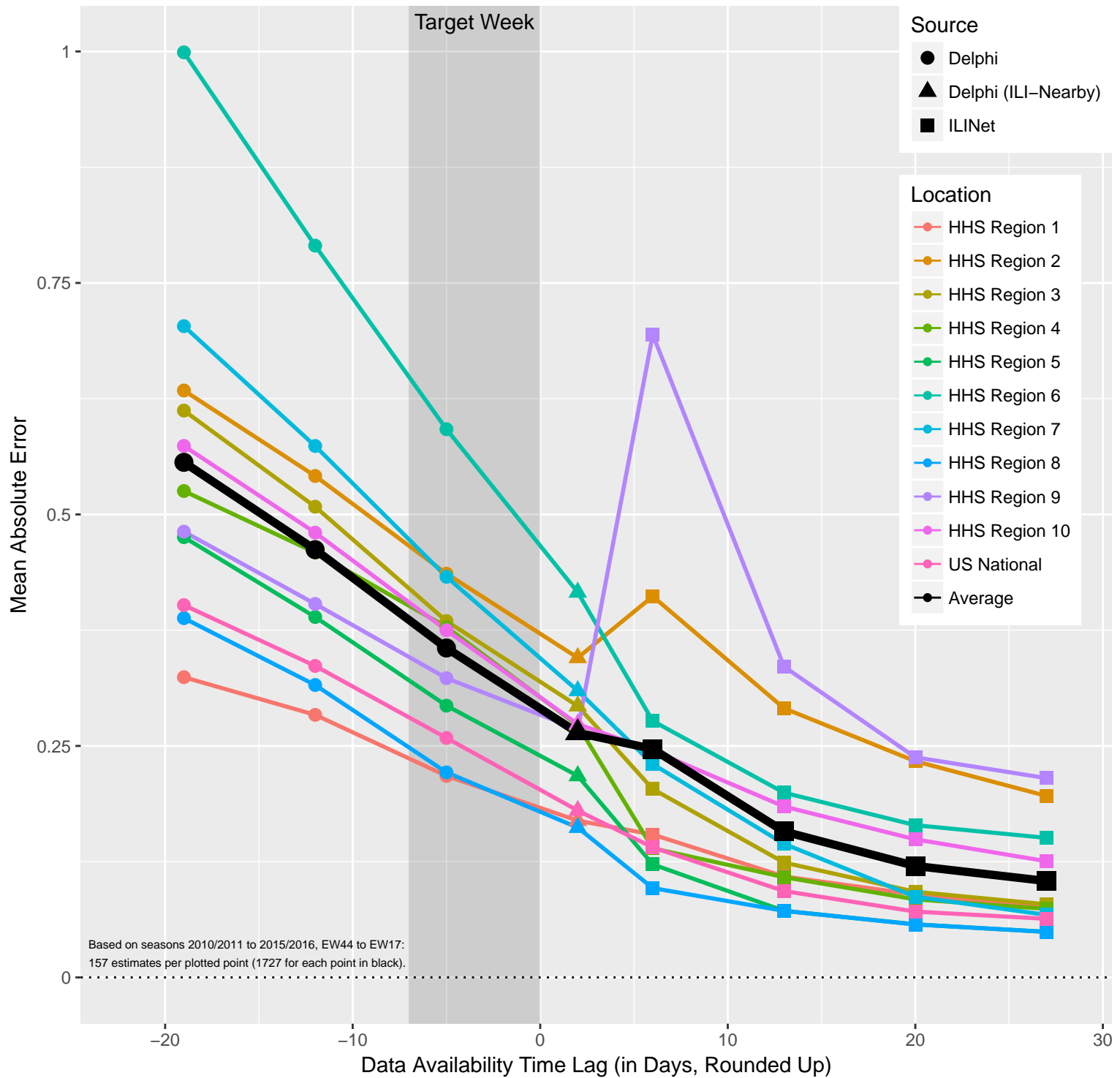

Supplement: S7 Fig — (PDF) [file pcbi.1006134.s007.pdf]

# Percent Error in wILI Estimates, Averaged across 10 HHS Regions + National

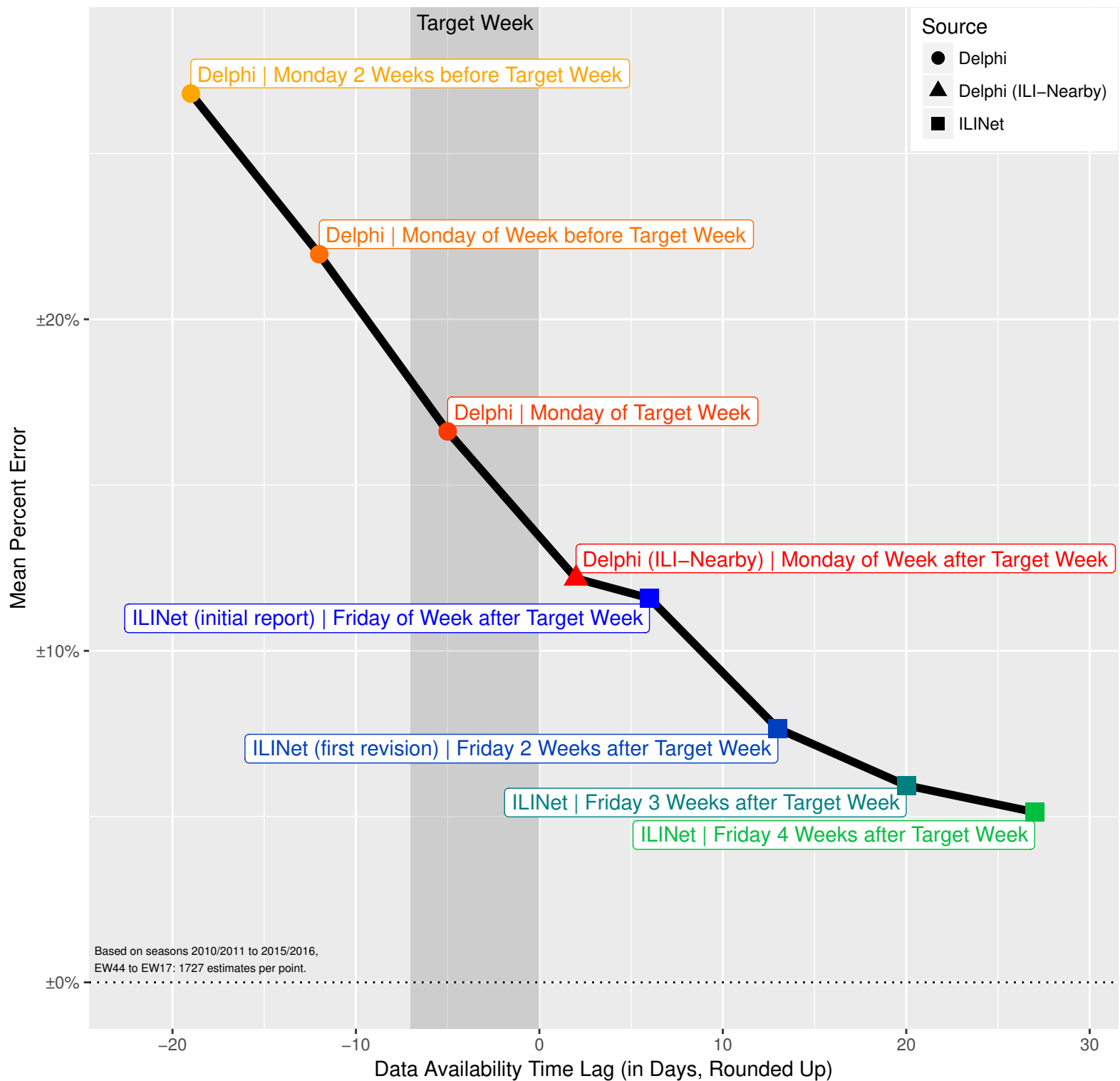

Supplement: S9 Fig — (PDF) [file pcbi.1006134.s009.pdf]

# Percent Error in wILI Estimates

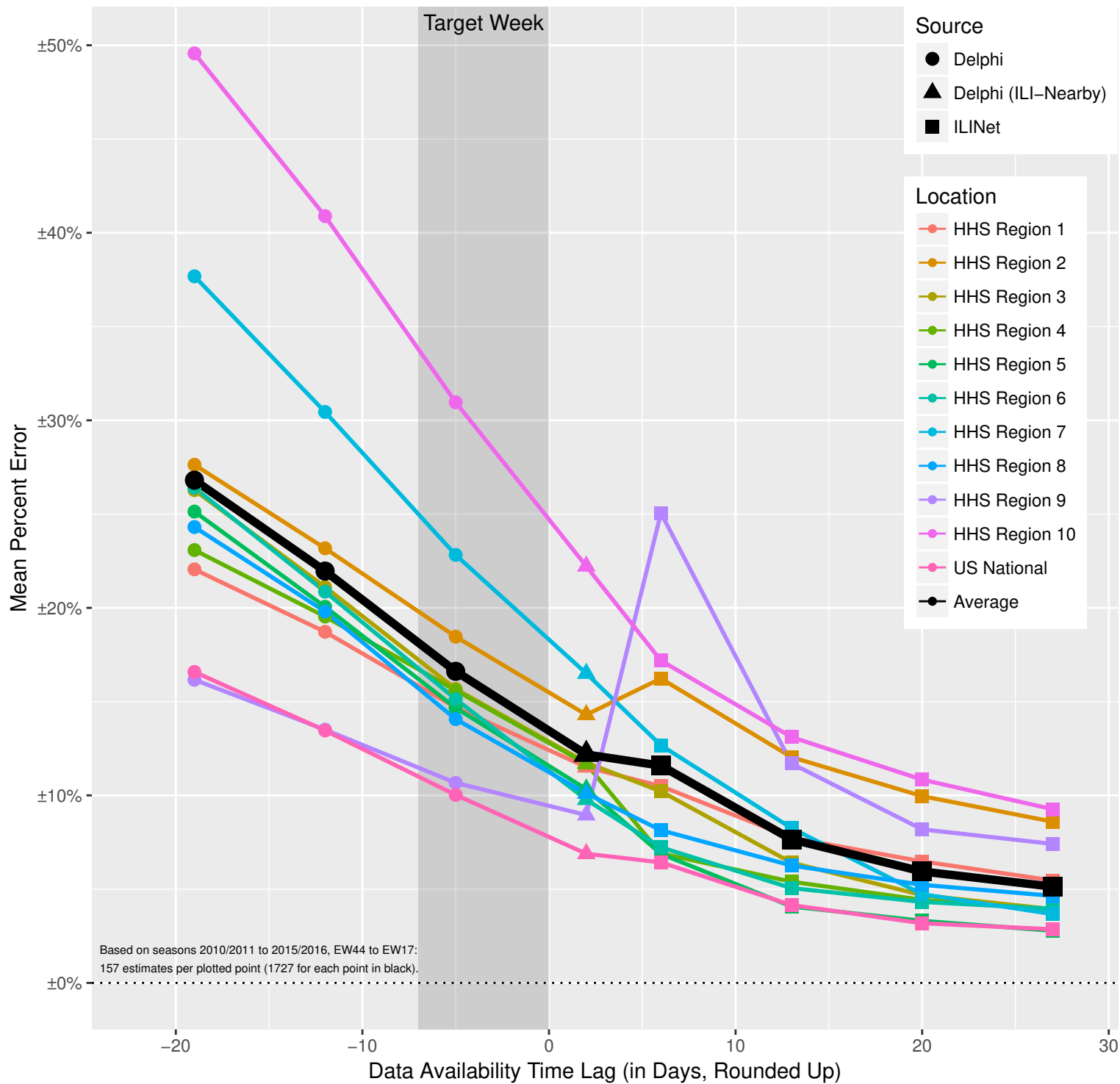

Supplement: S11 Fig — (PDF) [file pcbi.1006134.s011.pdf]
