# Supplementary material for: Nonmechanistic forecasts of seasonal influenza with iterative one-week-ahead distributions
Source: PLoS Comput Biol. 2018 Jun 15;14(6):e1006134. doi: 10.1371/journal.pcbi.1006134 (PMC6034894; doi:10.1371/journal.pcbi.1006134)
Supplement: S6 Fig — (PDF) [file pcbi.1006134.s006.pdf]

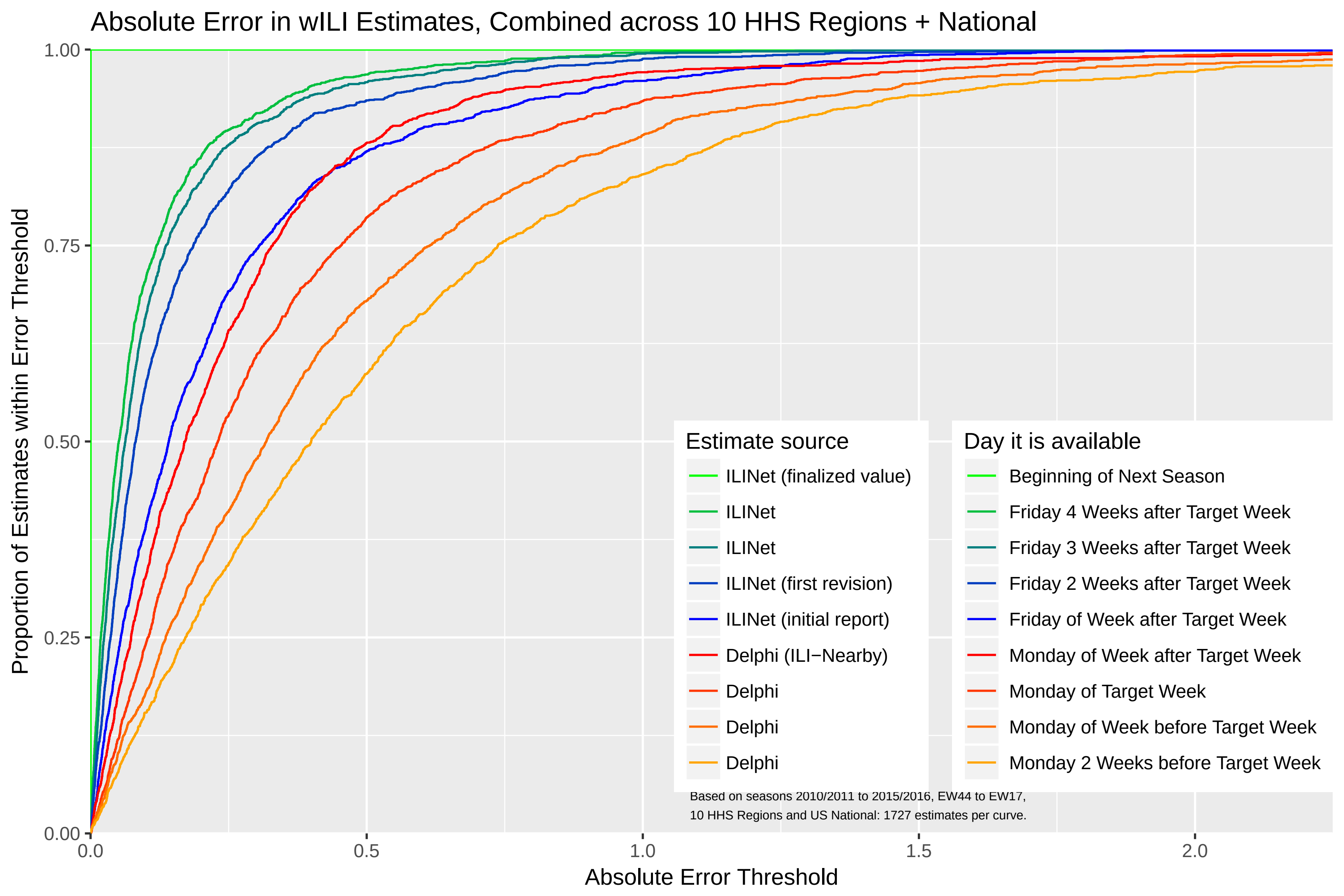

Estimate source	Day it is available	Mean Absolute Error
ILINet (finalized value)	Beginning of Next Season	0.00
ILINet	Friday 4 Weeks after Target Week	0.10
ILINet	Friday 3 Weeks after Target Week	0.12
ILINet (first revision)	Friday 2 Weeks after Target Week	0.16
ILINet (initial report)	Friday of Week after Target Week	0.25
Delphi (ILI−Nearby)	Monday of Week after Target Week	0.26
Delphi	Monday of Target Week	0.36
Delphi	Monday of Week before Target Week	0.46
Delphi	Monday 2 Weeks before Target Week	0.56
