# Supplementary material for: Nonmechanistic forecasts of seasonal influenza with iterative one-week-ahead distributions
Source: PLoS Comput Biol. 2018 Jun 15;14(6):e1006134. doi: 10.1371/journal.pcbi.1006134 (PMC6034894; doi:10.1371/journal.pcbi.1006134)
Supplement: S8 Fig — (PDF) [file pcbi.1006134.s008.pdf]

Absolute Error in wILI Estimates, by Location

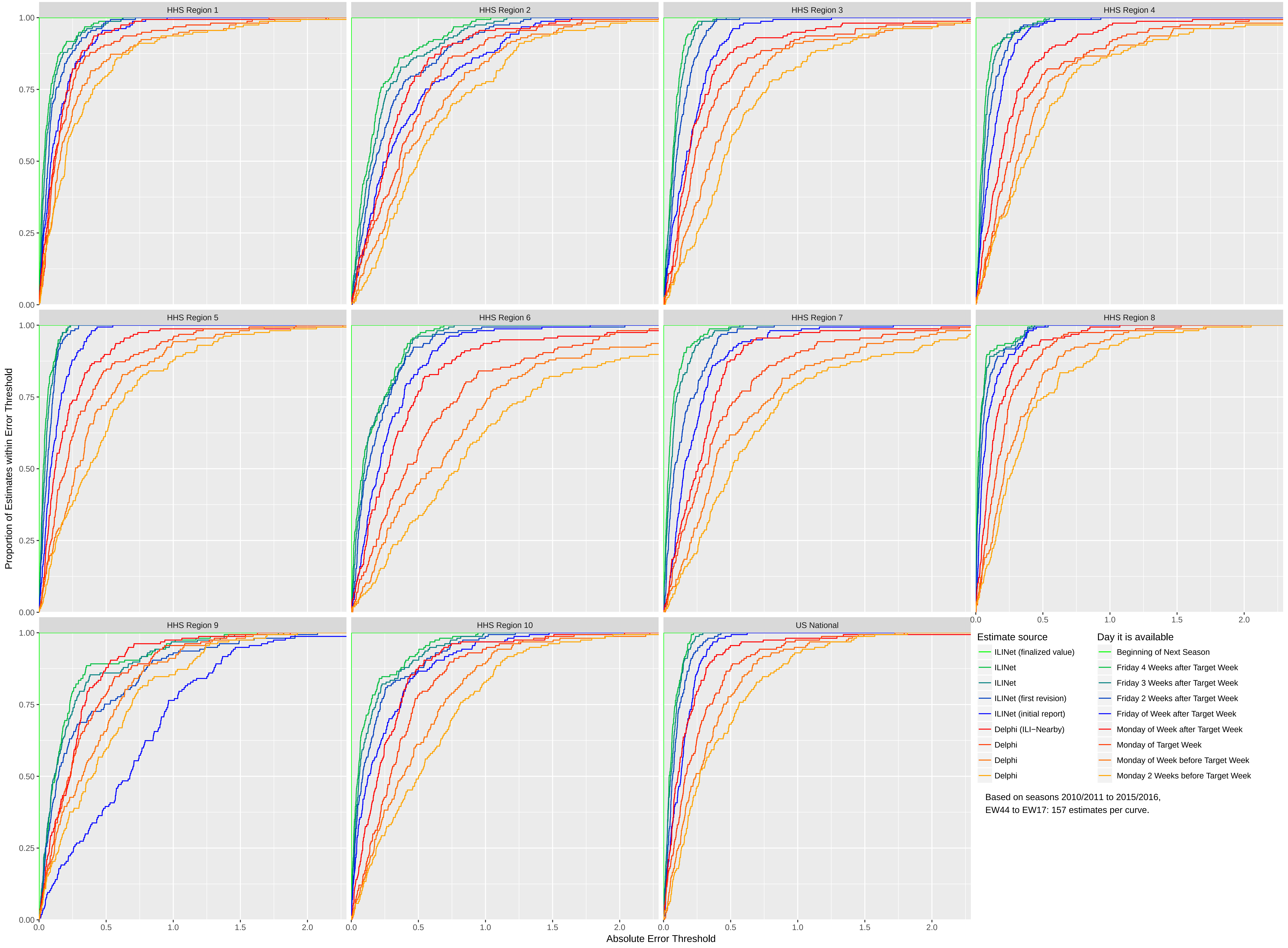

Estimate source	Day it is available	HHS Region 1	HHS Region 2	HHS Region 3	HHS Region 4	HHS Region 5	HHS Region 6	HHS Region 7	HHS Region 8	HHS Region 9	HHS Region 10	US National
ILINet (finalized value)	Beginning of Next Season	0.00	0.00	0.00	0.00	0.00	0.00	0.00	0.00	0.00	0.00	0.00
ILINet	Friday 4 Weeks after Target Week	0.08	0.20	0.08	0.07	0.05	0.15	0.07	0.05	0.22	0.13	0.06
ILINet	Friday 3 Weeks after Target Week	0.09	0.23	0.09	0.08	0.06	0.16	0.09	0.06	0.24	0.15	0.07
ILINet (first revision)	Friday 2 Weeks after Target Week	0.11	0.29	0.12	0.11	0.07	0.20	0.14	0.07	0.34	0.18	0.09
ILINet (initial report)	Friday of Week after Target Week	0.15	0.41	0.20	0.14	0.12	0.28	0.23	0.10	0.69	0.24	0.14
Delphi (ILI−Nearby)	Monday of Week after Target Week	0.17	0.35	0.29	0.27	0.22	0.42	0.31	0.16	0.27	0.27	0.18
Delphi	Monday of Target Week	0.22	0.44	0.38	0.38	0.29	0.59	0.43	0.22	0.32	0.37	0.26
Delphi	Monday of Week before Target Week	0.28	0.54	0.51	0.46	0.39	0.79	0.57	0.32	0.40	0.48	0.34
Delphi	Monday 2 Weeks before Target Week	0.32	0.63	0.61	0.53	0.48	1.00	0.70	0.39	0.48	0.57	0.40
