# Supplementary material for: Nonmechanistic forecasts of seasonal influenza with iterative one-week-ahead distributions
Source: PLoS Comput Biol. 2018 Jun 15;14(6):e1006134. doi: 10.1371/journal.pcbi.1006134 (PMC6034894; doi:10.1371/journal.pcbi.1006134)
Supplement: S10 Fig — (PDF) [file pcbi.1006134.s010.pdf]

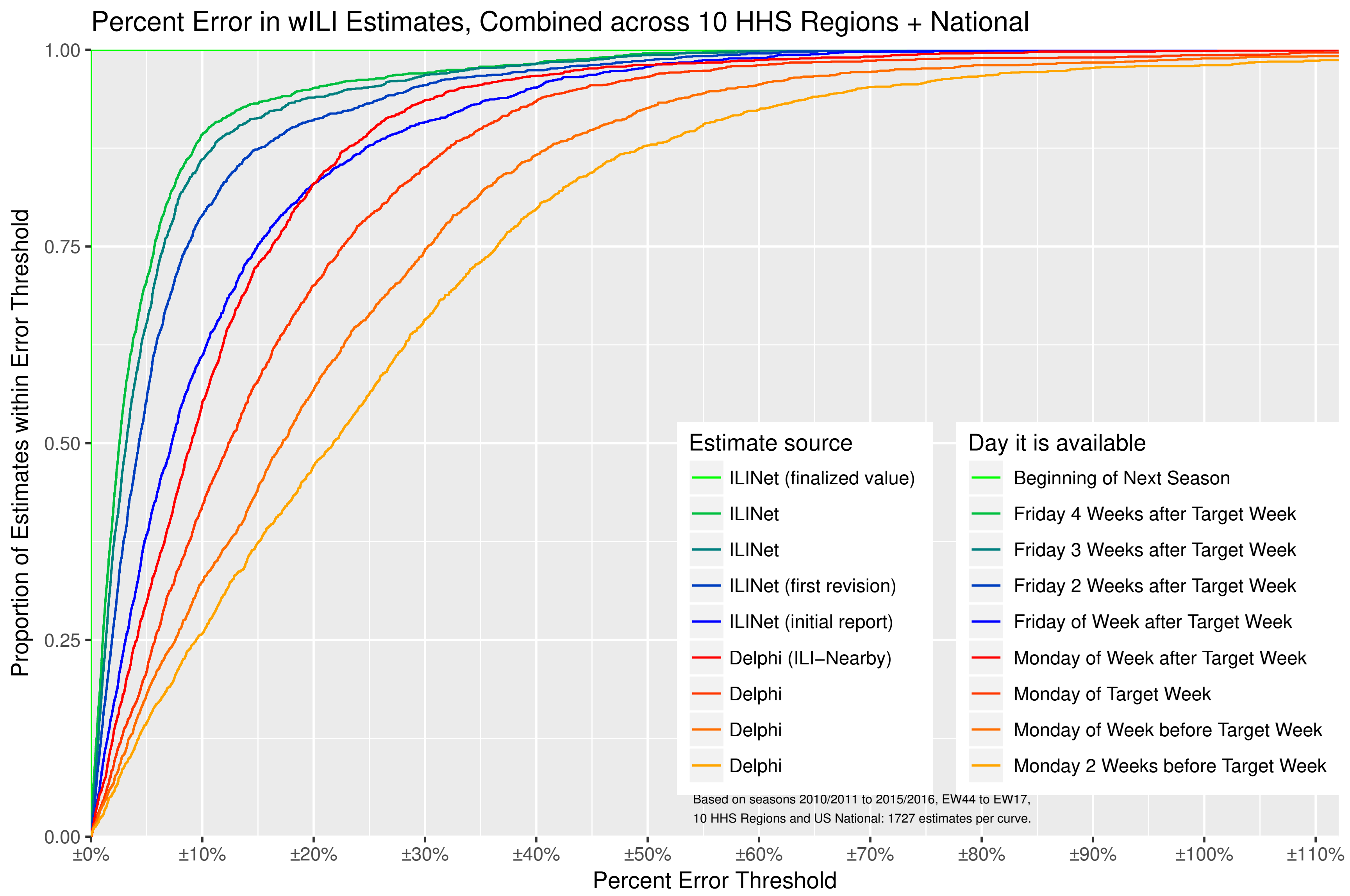

Estimate source	Day it is available	Mean Percent Error
ILINet (finalized value)	Beginning of Next Season	±0.0%
ILINet	Friday 4 Weeks after Target Week	±5.1%
ILINet	Friday 3 Weeks after Target Week	±5.9%
ILINet (first revision)	Friday 2 Weeks after Target Week	±7.7%
ILINet (initial report)	Friday of Week after Target Week	±11.6%
Delphi (ILI–Nearby)	Monday of Week after Target Week	±12.2%
Delphi	Monday of Target Week	±16.6%
Delphi	Monday of Week before Target Week	±22.0%
Delphi	Monday 2 Weeks before Target Week	±26.8%
