# Supplementary material for: Nonmechanistic forecasts of seasonal influenza with iterative one-week-ahead distributions
Source: PLoS Comput Biol. 2018 Jun 15;14(6):e1006134. doi: 10.1371/journal.pcbi.1006134 (PMC6034894; doi:10.1371/journal.pcbi.1006134)
Supplement: S12 Fig — (PDF) [file pcbi.1006134.s012.pdf]

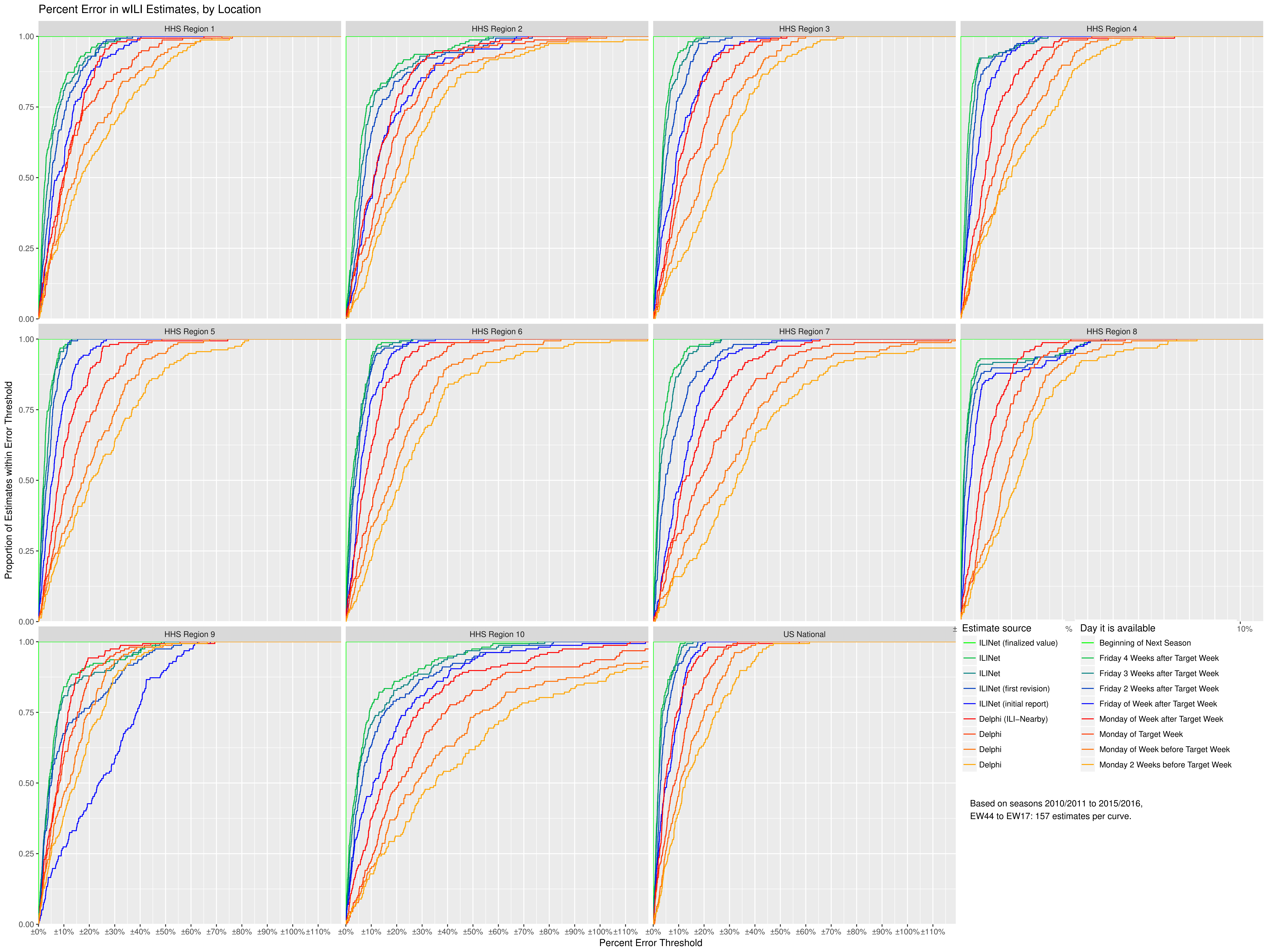

Estimate source	Day it is available	HHS Region 1	HHS Region 2	HHS Region 3	HHS Region 4	HHS Region 5	HHS Region 6	HHS Region 7	HHS Region 8	HHS Region 9	HHS Region 10	US National
ILINet (finalized value)	Beginning of Next Season	±0.0%	±0.0%	±0.0%	±0.0%	±0.0%	±0.0%	±0.0%	±0.0%	±0.0%	±0.0%	±0.0%
ILINet	Friday 4 Weeks after Target Week	±5.5%	±8.6%	±3.9%	±3.9%	±2.8%	±4.0%	±3.7%	±4.6%	±7.4%	±9.3%	±2.9%
ILINet	Friday 3 Weeks after Target Week	±6.5%	±10.0%	±4.7%	±4.4%	±3.3%	±4.3%	±4.7%	±5.2%	±8.2%	±10.8%	±3.2%
ILINet (first revision)	Friday 2 Weeks after Target Week	±7.7%	±12.0%	±6.4%	±5.4%	±4.1%	±5.1%	±8.3%	±6.3%	±11.7%	±13.1%	±4.2%
ILINet (initial report)	Friday of Week after Target Week	±10.5%	±16.2%	±10.2%	±6.9%	±6.9%	±7.2%	±12.7%	±8.1%	±25.1%	±17.2%	±6.4%
Delphi (ILI–Nearby)	Monday of Week after Target Week	±11.5%	±14.3%	±11.8%	±11.7%	±10.3%	±9.8%	±16.5%	±10.1%	±9.0%	±22.2%	±6.9%
Delphi	Monday of Target Week	±14.7%	±18.5%	±15.7%	±15.6%	±14.7%	±15.1%	±22.8%	±14.1%	±10.7%	±31.0%	±10.0%
Delphi	Monday of Week before Target Week	±18.7%	±23.2%	±21.1%	±19.5%	±20.1%	±20.9%	±30.4%	±19.8%	±13.5%	±40.9%	±13.5%
Delphi	Monday 2 Weeks before Target Week	±22.1%	±27.6%	±26.3%	±23.1%	±25.1%	±26.4%	±37.7%	±24.3%	±16.2%	±49.6%	±16.6%
