# Supplementary material for: Nonmechanistic forecasts of seasonal influenza with iterative one-week-ahead distributions
Source: PLoS Comput Biol. 2018 Jun 15;14(6):e1006134. doi: 10.1371/journal.pcbi.1006134 (PMC6034894; doi:10.1371/journal.pcbi.1006134)
Supplement: S1 Appendix — (PDF) [file pcbi.1006134.s013.pdf]

Description of all ensemble components in the 2015/2016 Delphi-Stat forecasting system

Ensemble components

Delphi-Stat incorporated 10 individual forecasting methods in the 2015/2016 season based on diverse methodologies to forecast the targets of interest $\mathbf{Z}^t$ conditioned on the finalized wILI values up to time t, $Y_{1..t}$. When producing prospective forecasts, we do not have access to the finalized values $Y_{1..t}$, but rather the t-th report for the current season, $W^t_{1..t}$; we discuss a method for distributional estimates of $Y_{1..t}$ based on $W^t_{1..t}$ in the main text. All methods produce distributional forecasts for the targets of interest; their point predictions are the medians of the corresponding distributional forecasts. Most methods, rather than directly producing forecasts for the targets, first estimate the distribution of the entire wILI trajectory $Y_{1..T}$ based on the available data, then calculates the corresponding distribution over the targets. (Since the data are at a weekly resolution, the number of wILI values in the current season, T, is either 52 or 53; we present the methods here as if all seasons were of the same length T, omitting all details dealing with mismatches between the length of a training season and the length of a test season.)

Methods based on delta density

Markovian delta density

Described in the main text.

Extended delta density

Described in the main text.

Methods based on empirical distribution of curves

Another class of methods are based on using and expanding the empirical distribution of wILI trajectories.

Empirical distribution of wILI trajectories for future times

Consider all $Y^s_{t+1..T}$, $s \in \{1 \,..\, S\}$, equally likely to reoccur. Observations from the current season are used for times up to t.

Empirical Bayes procedure on wILI trajectories

Model $Y_{1..T}$ as some underlying curve, $F_{1..T}$, plus i.i.d. Gaussian observational noise. Estimate $F^s_{1..T}$ and a noise level for each $s \in \{1 \,..\, S\}$ using a trend filtering procedure. Build a distribution for $F_{1..T}$ and the noise level using these estimates, plus a probability distribution over ways to shift and scale these

curves to produce a wider range of possibilities for $Y_{1..T}$. The resulting distribution describes our prior beliefs about the distribution of $Y_{1..T}$ before seeing any observations from the current season; calculate the corresponding posterior distribution, $Y_{1..T} \mid Y_{1..t}$, describing our beliefs after seeing the available observations, using importance sampling techniques [SAA1].

Implements the empirical Bayes method as described in [21], with a few modifications:

- Only the time-shift and wILI-scale transformations are used.
- The time-shift is a "local" transformation: rather than having a distribution of peak weeks determine the shift amount, we directly choose a distribution over shift amounts. Specifically, we use a discrete uniform distribution over integers centered at zero, width equal (ignoring rounding) to twice the bin width of a histogram of the historical peak weeks using Sturges' rule.
- The wILI-scale is a "local" transformation: rather than having a distribution of peak heights determine the scale amount, we directly choose a distribution over scale amounts. Specifically, we use a log-uniform distribution centered at 0 in the log-scale with log-scale width equal tot twice the bin width of a histogram of the logarithms of the historical peak heights, using Sturges' rule. Note that this behavior can significantly bias the mean of the prior for the peak heights, but does not significantly affect the median of the prior for the peak heights. Another difference from the scaling transformation in the paper is that, instead of scaling the wILI trajectory above and about the CDC baseline, we scale from 0, and also multiply the noise associated with each observation based on how much it was scaled.
- Instead of randomly mixing and matching smooth curve shapes and noise levels, these two parameters are linked together: a given noise level estimate is always paired with the corresponding smoothed curve.
- We add a "reasonable future" term to the posterior log-likelihood (given observations in past weeks) of each proposed trajectory, proportional to the average log-likelihood of the 3 most similar historical curves in future weeks.
- We condition on a maximum of 5 observations from the current season; if more than 5 observations are available for the current season, we use only the most recent 5.
- We use the `glmgen` package [SAA2] to rapidly perform trend filtering for smoothing past seasons' trajectories.

We form two other versions of the empirical Bayes forecaster by using subsets of these changes and other parameter settings; these variants were used in the 2016/2017 ensemble but not the 2015/2016 ensemble.

Basis regression approach

Estimates the mean curve $\mathbb{E}Y_{1..T}$ with elastic net regression from a collection of basis functions to a trajectory of "pseudo-observations" $\tilde{Y}_{1..T}$ which is the concatenation of (a) the available observations $Y_{1..t}$, and (b) the pointwise mean of $Y^s_{t+1..T}$ for $s \in \{1 .. S\}$. We chose a B-spline basis, which produces a variation on smoothing spline estimation of $\mathbb{E}Y_{1..T}$. The `glmnet` package [SAA3] was used to perform the elastic net regression, with evenly weighed L^1 and L^2 regularization (the default setting, $\alpha = 0.5$), and to automatically select the overall regression penalty coefficient λ using random 5-fold cross-validation on weeks of the current season, seeing how well the smoothed estimate for $\mathbb{E}Y_{1..T}$ is able to predict left-out pseudo-observations from $\tilde{Y}_{1..T}$.

Basis regression with degenerate distributional forecast

Forecasts that $Y_{1..T}$ will be equal to the basis regression estimate for $\mathbb{E}Y_{1..T}$ with probability 1. There is a small amount of randomness in the basis regression estimation procedure itself arising from the default method for selecting λ, so we actually take a sample by calling the procedure many times, forming a very narrow distribution.

Basis regression with residual density distributional forecast

Constructs a distributional forecast for $Y_{1..T}$ by applying the residual density method with $X_{1..T}$ equal to the basis regression estimate for $\mathbb{E}Y_{1..T}$ and other settings the same as in the Markovian delta density method.

No-trajectory approaches

These approaches form a forecast for $\mathbf{Z}^t$ from an estimate of $Y_{1..t}$ without first constructing a forecast for the entire trajectory $Y_{1..T}$.

Empirical distribution of target values

Consider all $Y^s_{1..T}$, $s \in \{1 .. S\}$, equally likely to reoccur, ignoring and overriding the available observations from the current season ($Y_{1..t}$). For each target, the distributional forecast is its empirical distribution, and the point prediction is the corresponding median.

Direct target forecasts with kernel smoothing

Uses the kernel smoothing method used in the delta density method to estimate the distribution of $\mathbf{Z}^t$ conditioned only on (an estimate of) Y_t.

Direct target forecasts with generalized additive model

Uses a generalized additive model to predict the expected value of a subset of the targets, and assumes a normal distribution for the residuals when making

distributional forecasts. Provided by Shannon Gallagher. This method was used in the 2015/2016 ensemble, but not the 2016/2017 ensemble nor the cross-validation analysis.

Uniform distribution

Outputs the same probability for each bin, regardless of the input data. The corresponding point predictions are excluded from the ensemble.

Supplementary references

[SAA1] Liu JS. Monte Carlo strategies in scientific computing. Springer Science & Business Media; 2008.

[SAA2] Arnold T, Sadhanala V, Tibshirani R. glmgen: Fast algorithms for generalized lasso problems; 2014.

[SAA3] Friedman J, Hastie T, Tibshirani R. Regularization Paths for Generalized Linear Models via Coordinate Descent. Journal of Statistical Software. 2010;33(1):1–22.
