# Supplementary material for: Nonmechanistic forecasts of seasonal influenza with iterative one-week-ahead distributions
Source: PLoS Comput Biol. 2018 Jun 15;14(6):e1006134. doi: 10.1371/journal.pcbi.1006134 (PMC6034894; doi:10.1371/journal.pcbi.1006134)
Supplement: S2 Appendix — (PDF) [file pcbi.1006134.s014.pdf]

Tabular evaluations of 2015/2016 forecasts

Metric	Target	#Obs.	Forecasting System		
			Delphi-Archefilter	Delphi-Epicast	Delphi-Stat
Multibin log score	Overall	2233	-0.95 [0.390]	-0.86 [0.420]	**-0.81 [0.440]**
	Season onset	319	**-1.69 [0.180]**	-1.98 [0.140]	**-1.62 [0.200]**
	Season peak week	319	**-1.45 [0.230]**	-1.51 [0.220]	**-1.39 [0.250]**
	Season peak percentage	319	-0.73 [0.480]	**-0.60 [0.550]**	-0.66 [0.520]
	1 wk ahead	319	-0.53 [0.590]	**-0.33 [0.720]**	**-0.33 [0.720]**
	2 wk ahead	319	-0.65 [0.520]	**-0.42 [0.660]**	-0.47 [0.630]
	3 wk ahead	319	-0.75 [0.470]	**-0.52 [0.590]**	-0.57 [0.560]
	4 wk ahead	319	-0.86 [0.420]	**-0.64 [0.530]**	**-0.64 [0.530]**
Unibin log score	Overall	2233	-2.01 [0.130]	-1.92 [0.150]	**-1.74 [0.180]**
	Season onset	319	-2.73 [0.070]	-2.94 [0.050]	**-2.42 [0.090]**
	Season peak week	319	-2.57 [0.080]	-2.45 [0.090]	**-2.14 [0.120]**
	Season peak percentage	319	**-1.73 [0.180]**	**-1.74 [0.170]**	**-1.69 [0.180]**
	1 wk ahead	319	-1.55 [0.210]	-1.30 [0.270]	**-1.24 [0.290]**
	2 wk ahead	319	-1.70 [0.180]	**-1.45 [0.230]**	**-1.42 [0.240]**
	3 wk ahead	319	-1.83 [0.160]	-1.68 [0.190]	**-1.56 [0.210]**
	4 wk ahead	319	-1.96 [0.140]	-1.90 [0.150]	**-1.68 [0.190]**
Absolute Error	Overall	2233	1.10	**1.05**	1.11
	Season onset	319	**2.59**	**2.56**	**2.64**
	Season peak week	319	**2.40**	2.57	2.84
	Season peak percentage	319	0.60	**0.49**	0.57
	1 wk ahead	319	0.39	**0.31**	0.33
	2 wk ahead	319	0.48	**0.38**	0.42
	3 wk ahead	319	0.58	**0.46**	**0.47**
	4 wk ahead	319	0.66	0.55	**0.52**

Table A: **The three Delphi systems had similar overall scores; Delphi-Stat gave the best distributional forecasts overall, while Delphi-Epicast gave the best point predictions overall.** This table contains evaluations for the 2015/2016 season, averaged across locations and forecast weeks, for each target and a few evaluation metrics. Higher log scores and lower absolute error indicate better performance on distributional forecasts and point predictions, respectively. Values within 5% of the best value in each row are typeset in boldface. Numbers in brackets are the exponentiation of the corresponding log score. Each entry for a specific target is an average of 319 evaluations (from forecasts for 11 locations made over 29 weeks), giving a total of 2233 evaluations overall for each system.

Grouping variable	Group value	#Obs.	Forecasting System		
			Delphi-Archefilter	Delphi-Epicast	Delphi-Stat
Location	HHS Region 1	203	-0.76 [0.470]	**-0.60 [0.550]**	**-0.59 [0.560]**
	HHS Region 2	203	**-1.39 [0.250]**	-1.56 [0.210]	**-1.42 [0.240]**
	HHS Region 3	203	-0.70 [0.490]	**-0.60 [0.550]**	-0.69 [0.500]
	HHS Region 4	203	-1.21 [0.300]	-1.32 [0.270]	**-1.04 [0.350]**
	HHS Region 5	203	-0.86 [0.420]	-0.80 [0.450]	**-0.65 [0.520]**
	HHS Region 6	203	-1.56 [0.210]	**-0.77 [0.460]**	-0.85 [0.430]
	HHS Region 7	203	-1.03 [0.360]	-1.00 [0.370]	**-0.91 [0.400]**
	HHS Region 8	203	-0.52 [0.600]	-0.49 [0.620]	**-0.41 [0.660]**
	HHS Region 9	203	**-0.65 [0.520]**	-0.72 [0.490]	-0.73 [0.480]
	HHS Region 10	203	-0.76 [0.470]	**-0.56 [0.570]**	-0.74 [0.480]
	US National	203	-1.04 [0.350]	-1.03 [0.360]	**-0.90 [0.410]**
Forecast Week	1	77	-1.07 [0.340]	**-0.94 [0.390]**	-1.07 [0.340]
	2	77	-0.99 [0.370]	**-0.82 [0.440]**	-1.02 [0.360]
	3	77	-0.99 [0.370]	**-0.79 [0.460]**	-0.98 [0.380]
	4	77	-0.92 [0.400]	**-0.83 [0.440]**	-0.94 [0.390]
	5	77	**-0.84 [0.430]**	-0.98 [0.380]	-0.90 [0.410]
	6	77	**-0.86 [0.420]**	-0.94 [0.390]	**-0.82 [0.440]**
	7	77	-0.85 [0.430]	-0.89 [0.410]	**-0.74 [0.480]**
	8	77	-0.74 [0.480]	-0.87 [0.420]	**-0.64 [0.520]**
	9	77	-0.65 [0.520]	-0.50 [0.610]	**-0.47 [0.630]**
	10	77	-0.58 [0.560]	**-0.33 [0.720]**	-0.38 [0.680]
	11	77	-0.53 [0.590]	**-0.32 [0.720]**	-0.39 [0.680]
	12	77	-0.56 [0.570]	-0.40 [0.670]	**-0.37 [0.690]**
	13	77	-0.49 [0.610]	-0.30 [0.740]	**-0.28 [0.750]**
	14	77	-0.45 [0.640]	-0.27 [0.760]	**-0.26 [0.770]**
	15	77	-0.39 [0.670]	**-0.22 [0.810]**	-0.24 [0.790]
	16	77	-0.37 [0.690]	**-0.19 [0.830]**	-0.21 [0.810]
	17	77	-0.36 [0.700]	**-0.19 [0.820]**	-0.23 [0.790]
	18	77	-0.37 [0.690]	**-0.21 [0.810]**	**-0.22 [0.810]**
	42	77	-1.38 [0.250]	-1.31 [0.270]	**-1.05 [0.350]**
	43	77	-1.19 [0.300]	-1.17 [0.310]	**-1.03 [0.360]**
	44	77	-1.15 [0.320]	-1.18 [0.310]	**-1.03 [0.360]**
	45	77	**-1.14 [0.320]**	-1.25 [0.290]	**-1.13 [0.320]**
	46	77	-1.23 [0.290]	-1.43 [0.240]	**-1.15 [0.320]**
	47	77	-1.52 [0.220]	-1.58 [0.210]	**-1.29 [0.270]**
	48	77	-1.82 [0.160]	-1.39 [0.250]	**-1.31 [0.270]**
	49	77	-1.82 [0.160]	-1.52 [0.220]	**-1.44 [0.240]**
	50	77	-1.62 [0.200]	**-1.20 [0.300]**	-1.37 [0.250]
	51	77	**-1.42 [0.240]**	**-1.39 [0.250]**	**-1.37 [0.250]**
	52	77	-1.31 [0.270]	-1.47 [0.230]	**-1.21 [0.300]**

Table B: **Delphi-Stat had consistently good aggregate multibin log score for different locations and weeks.** This table contains average multibin log scores for the 2015/2016 season, including all targets, broken down by location and forecast week. Each entry for a specific location is an average of 203 evaluations, and each entry for a specific forecast week is an average of 77 evaluations. Higher log scores indicate better performance of distributional forecasts. Values within 5% of the best log score in each row are typeset in boldface. Numbers in brackets are the exponentiation of the corresponding log score.
