# Supplementary material for: Nonmechanistic forecasts of seasonal influenza with iterative one-week-ahead distributions
Source: PLoS Comput Biol. 2018 Jun 15;14(6):e1006134. doi: 10.1371/journal.pcbi.1006134 (PMC6034894; doi:10.1371/journal.pcbi.1006134)
Supplement: S3 Appendix — (PDF) [file pcbi.1006134.s015.pdf]

“Missed possibilities” and -10 log score threshold

Individual unibin and multibin log scores below -10 have been increased to a minimum of -10 (as if a probability of ≈ 0.0000454 — still very small for the selected bin sizes — had been assigned) in all analysis. This threshold operation can be interpreted as adding up to a certain amount of probability mass to a distributional forecast, which normally has a total probability mass of 1; the maximum number of bins in any target's distributional forecast is 131, so the threshold operation cannot increase the amount of probability mass to more than ≈ 1.006. This threshold was implemented by CDC for forecast comparisons so that submissions would not be assigned very low mean log scores (e.g., $-\infty$) for assigning a few events extremely low (e.g., 0) probabilities to events that actually occurred. We also use it when comparing individual methods in the ensemble. Without such a threshold, each FluSight submission or ensemble component would need to ensure that no possibilities are missed and assigned extremely low probabilities, e.g., by mixing model forecasts with a uniform distribution (which bears similarity to the threshold operation) using the rule of three to determine the mixing weights. Thresholded log scores are no longer proper scores, as forecasters may expect to benefit by reporting probabilities of 0 for any bin with a modeled probability less than the exponentiated threshold, and using the difference in mass to increase probabilities assigned to other bins; with a threshold of -10, there is not much expected benefit (at most ≈ 0.006 mass would be reassigned), but at higher thresholds, this impropriety may be problematic. The stacking-based ensembles presented in the main text, and in this appendix unless otherwise noted, use weight selections intended to maximize mean unibin log score without thresholding.

For the full Delphi-Stat ensemble, the main advantage of the ensemble over its best component appears to be successfully filling in possibilities missed by the best component with other models to avoid -10 and other low log scores appears, while for ensembles of subsets of the forecasting methods, there are other benefits. We investigate changes to this log score threshold, and experiment with removing the lowest $p\%$ of log scores instead. As the log score threshold or p is increased, the relative performance of an ensemble over the best component declines and becomes negative when the ensemble is still tuned to optimize non-thresholded log score. Tailoring the optimization criterion to better match modified evaluation criteria can help restore the ensemble's superior or competitive performance compared to its best component.

Analysis of full Delphi-Stat ensemble

Fig A shows histograms of the cross validation log scores of the Delphi-Stat components and full ensemble with the original $-10 \approx \log(0.0000454)$ threshold; compared with the extended delta density method, the adaptively weighted ensemble:

- has higher mean log score;

- eliminates all -10 log scores;
- has less log scores of 0, but more right below 0; and
- smoother and wider tails about the mode of the histogram near the mean log score.

Fig B shows the same histograms using a threshold of $-7 \approx \log(0.000912)$; the four points above still hold, but the difference in mean log score between the two forecasters is notably smaller.

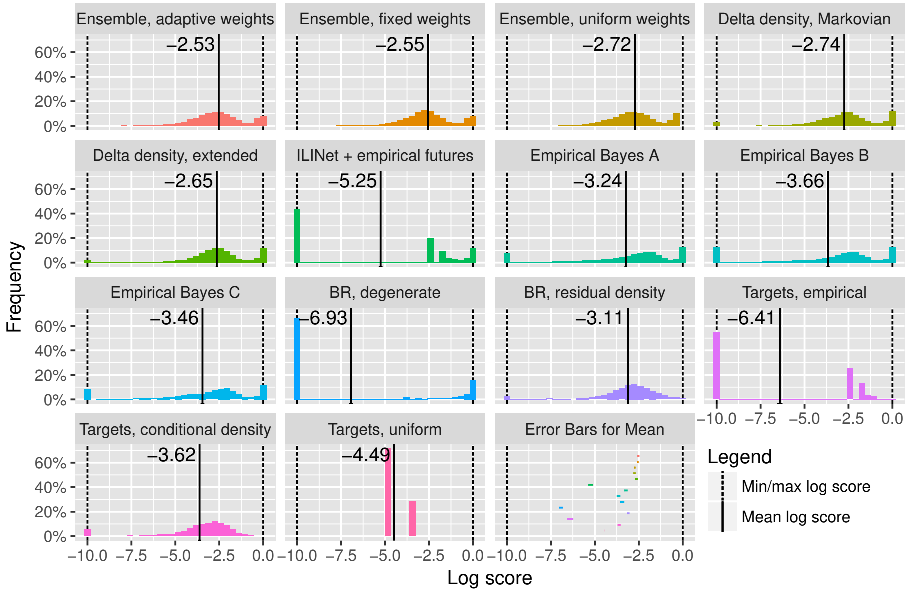

Fig A: **Fig 5 from the main text: log score means and histograms for each method using a log score threshold of -10, and ensemble weights trained ignoring the log score threshold.** This figure contains histograms of cross-validation log scores for a variety of forecasting methods, averaged across seasons 2010/2011 to 2015/2016, all locations, forecast weeks 40 to 20, and all forecasting targets. The solid black vertical lines indicate the mean of the scores in each histogram, which we use as the primary figure of merit when comparing forecasting methods; a rough error bar for each of these mean scores is shown as a colored horizontal bar in the last panel, and as a black horizontal line at the bottom of the corresponding histogram if the error bar is wider than the thickness of the black vertical line.

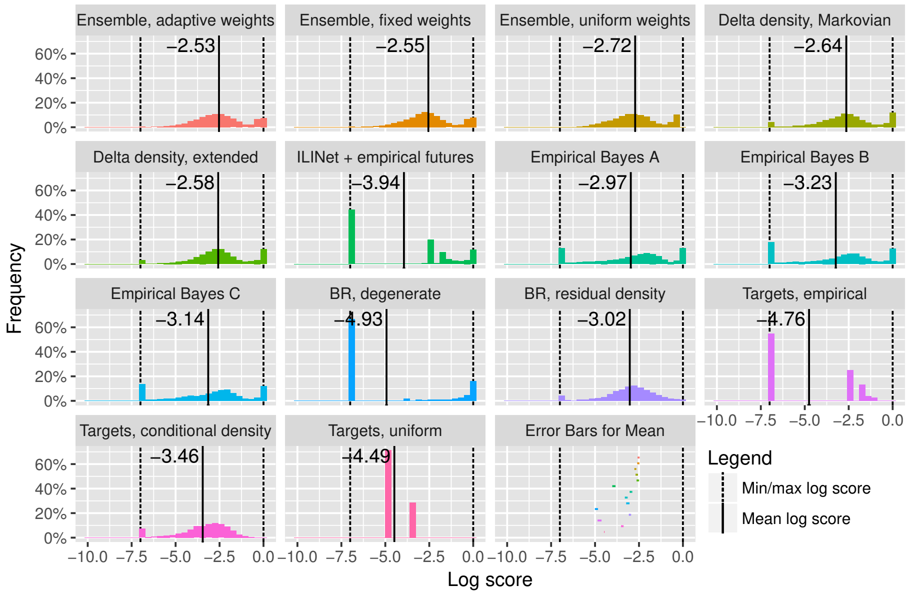

Fig B: **Log score means and histograms for each method using a log score threshold of -7 and ensemble weights trained ignoring the log score threshold.**

Fig C and Fig D show that the adaptively weighted ensemble and extended delta density are surpassed by other methods for thresholds from -3 to 0. However, Fig D also shows that a threshold of $-3 \approx \log(0.0498)$ already changes from 25% to over 50% of the log scores for each method, which seems inappropriate. Nevertheless, ensemble methods could still be useful in this case, but the weight selection objective must be updated to better match the evaluation metric; Fig E shows that the ensemble score can be improved significantly by solving a relaxation (approximation) of the thresholded log score optimization problem.

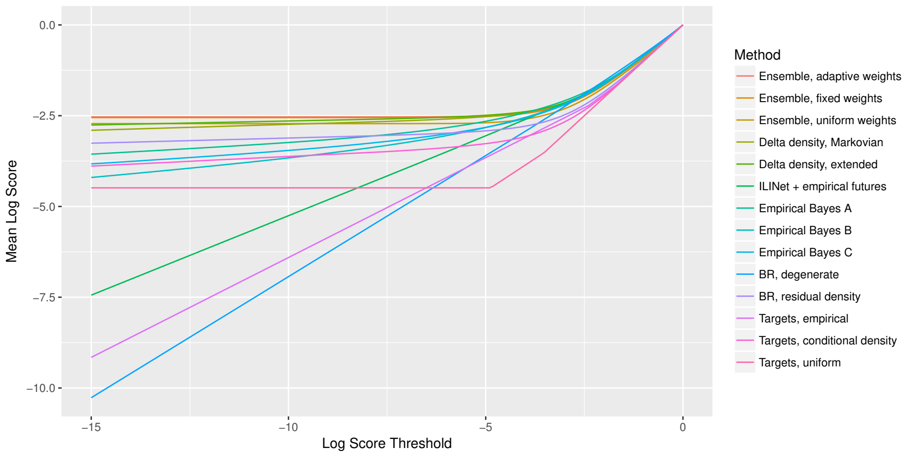

Fig C: **Thresholded mean log scores for each method and thresholds from -15 to 0.**

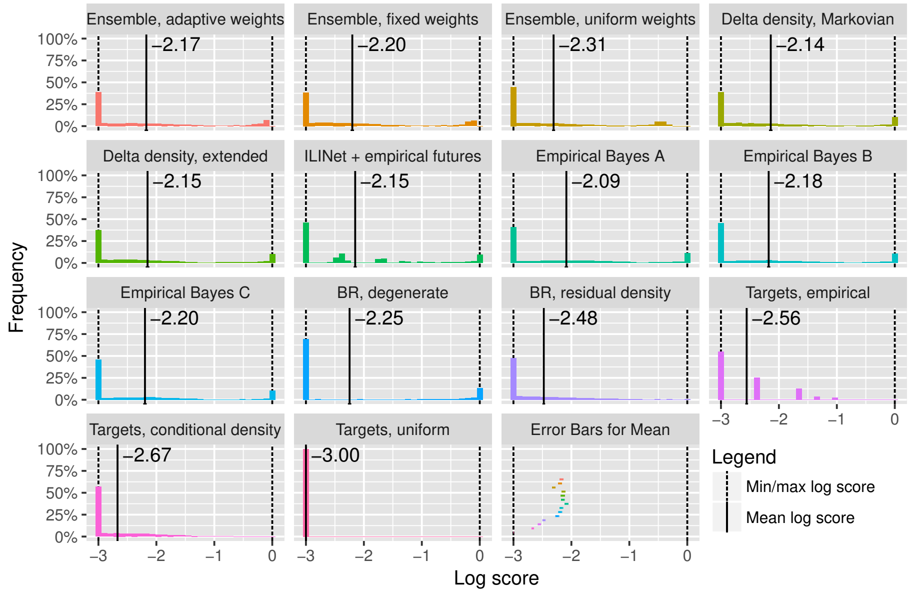

Fig D: **Log score means and histograms for each method using a log score threshold of -3 and ensemble weights trained ignoring the log score threshold.** Note that the ranges of values shown along both axes differ from the ranges used for similar figures for the -10 and -7 thresholds.

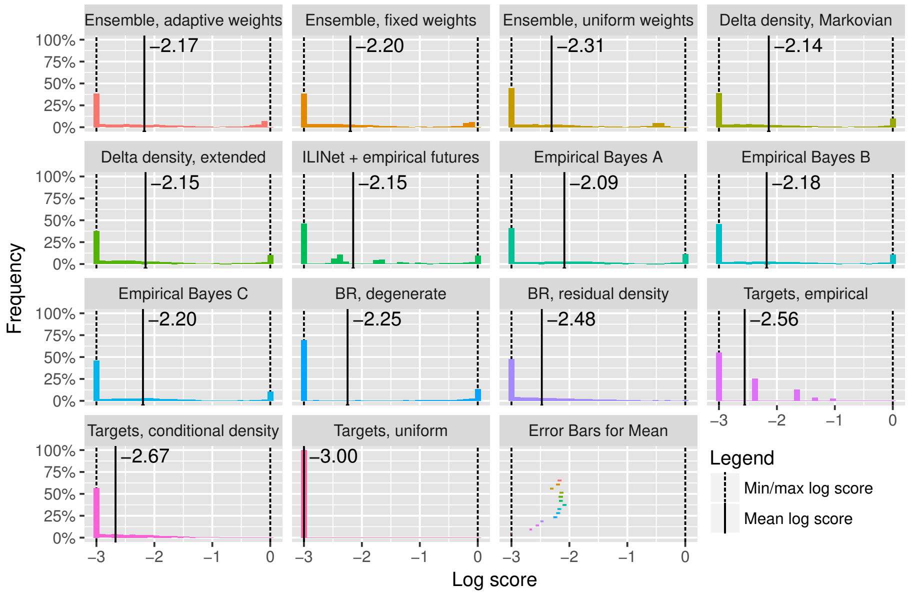

Fig E: **Log score means and histograms for each method using a log score threshold of -3 and ensemble weights trained using a concave relaxation of thresholded log score.** Note that the ranges of values shown along both axes differ from the ranges used for similar figures for the -10 and -7 thresholds.

The relative trends are similar when throwing away the lowest $p\%$ of log scores for a method rather than imposing a minimum log score threshold; Fig F shows that, when p is high enough to discard all $-\infty$ log scores for delta density methods, their performance is similar to that of the ensemble. Again, optimizing the ensemble weights to these modified error metrics could potentially result in performance improvements.

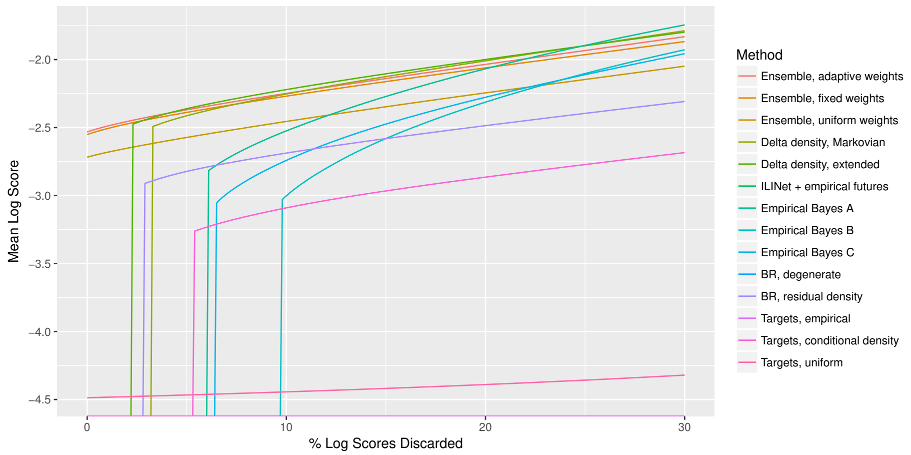

Fig F: **Mean log score for each method in the full ensemble, with no thresholding but throwing out the lowest p percent of log scores for each method for various values of p.**

Analysis of subset of methods

Fig G shows log score histograms for a subset of the methods above and ensembles using only those methods. The best component in this subset is "Targets, conditional density", which is completely missing the spike in log scores near 0 present in "Empirical Bayes B" and "BR, degenerate" (which model trajectories and calculate target distributions from these trajectory distributions), but still has higher mean log score than these two due to less scores of -10 and a higher concentration of scores from -5 to -1. The ensemble is able to combine the strengths of these models and the uniform distribution, avoiding any scores of -10 (or even -8), incorporating a spike in log scores near 0, and concentrating the rest of its log scores on the higher end of the -8 to -1 range. "Empirical Bayes B" is a close second to "Targets, conditional density", but the ensemble approach provides additional benefit besides just avoiding its missed possibilities; Fig H shows that, even when ignoring the lowest 10% of log scores for each method (which removes all scores of $-\infty$ for "Empirical Bayes B"), the adaptively weighted ensemble provides a large improvement in log score. This benefit vanishes and "Empirical Bayes B" starts to perform better as higher percentages (20% to 30%) of log scores are ignored; again, it may be possible to construct a successful ensemble in these cases by choosing an optimization criterion more similar to the evaluation criterion.

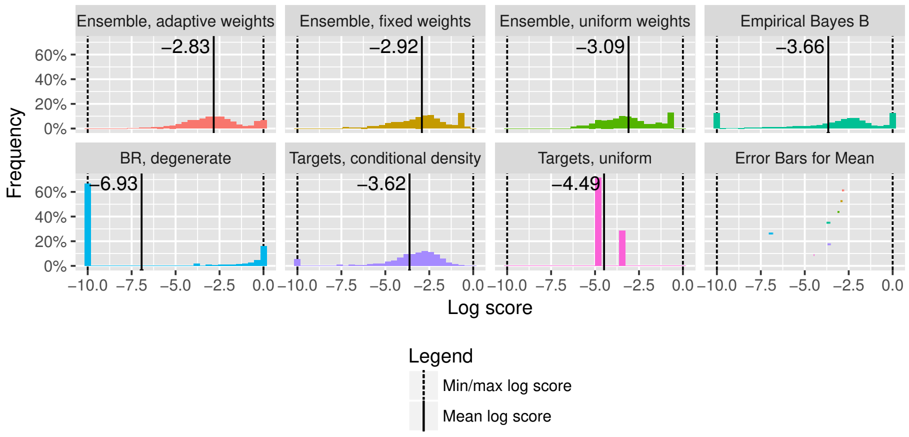

Fig G: **Log score means and histograms for a subset of methods (the same as the subset in Fig 6 of the main text) using a log score threshold of -10, and ensemble weights trained ignoring the log score threshold.**

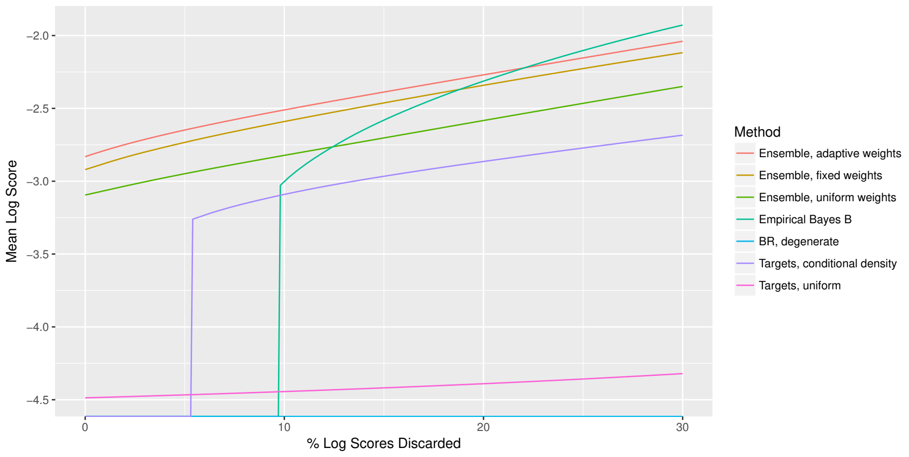

Fig H: **Mean log score for each method in the subset ensemble, with no thresholding but throwing out the lowest p percent of log scores for each method for various values of p.**
