# Supplementary material for: Nonmechanistic forecasts of seasonal influenza with iterative one-week-ahead distributions
Source: PLoS Comput Biol. 2018 Jun 15;14(6):e1006134. doi: 10.1371/journal.pcbi.1006134 (PMC6034894; doi:10.1371/journal.pcbi.1006134)
Supplement: S4 Appendix — (PDF) [file pcbi.1006134.s016.pdf]

Tabular evaluations of CV forecasts

Metric	Target	Method	Mean	Rough Standard Error Of Mean
Unibin log score	Season onset	Ensemble, adaptive weights (AWE)	-1.43	0.11
Unibin log score	Season onset	Ensemble, fixed weights	-1.45	0.11
Unibin log score	Season onset	Ensemble, uniform weights	-1.60	0.09
Unibin log score	Season onset	Delta density, Markovian	-1.54	0.17
Unibin log score	Season onset	Delta density, extended (EDD)	-1.62	0.18
Unibin log score	Season onset	ILINet + empirical futures	-2.51	0.25
Unibin log score	Season onset	Empirical Bayes A (EBA)	-2.18	0.20
Unibin log score	Season onset	Empirical Bayes B	-2.33	0.23
Unibin log score	Season onset	Empirical Bayes C	-2.37	0.25
Unibin log score	Season onset	BR, degenerate	-3.74	0.28
Unibin log score	Season onset	BR, residual density	-2.32	0.20
Unibin log score	Season onset	Targets, empirical	-5.56	0.49
Unibin log score	Season onset	Targets, conditional density	-3.53	0.25
Unibin log score	Season onset	Targets, uniform (UNIF)	-3.53	0.00
Unibin log score	Season onset	AWE improvement on EDD	0.18	0.08
Unibin log score	Season onset	EDD improvement on EBA	0.57	0.12
Unibin log score	Season onset	EBA improvement on UNIF	1.35	0.20
Unibin log score	Season peak week	Ensemble, adaptive weights (AWE)	-1.67	0.11
Unibin log score	Season peak week	Ensemble, fixed weights	-1.66	0.11
Unibin log score	Season peak week	Ensemble, uniform weights	-1.81	0.09
Unibin log score	Season peak week	Delta density, Markovian	-1.84	0.17
Unibin log score	Season peak week	Delta density, extended (EDD)	-1.73	0.15
Unibin log score	Season peak week	ILINet + empirical futures	-2.99	0.31
Unibin log score	Season peak week	Empirical Bayes A (EBA)	-2.42	0.14
Unibin log score	Season peak week	Empirical Bayes B	-2.60	0.18
Unibin log score	Season peak week	Empirical Bayes C	-2.48	0.20
Unibin log score	Season peak week	BR, degenerate	-5.07	0.21
Unibin log score	Season peak week	BR, residual density	-2.42	0.18
Unibin log score	Season peak week	Targets, empirical	-5.07	0.51
Unibin log score	Season peak week	Targets, conditional density	-3.31	0.19
Unibin log score	Season peak week	Targets, uniform (UNIF)	-3.50	0.00
Unibin log score	Season peak week	AWE improvement on EDD	0.05	0.04
Unibin log score	Season peak week	EDD improvement on EBA	0.69	0.14
Unibin log score	Season peak week	EBA improvement on UNIF	1.08	0.14
Unibin log score	Season peak percentage	Ensemble, adaptive weights (AWE)	-3.04	0.09
Unibin log score	Season peak percentage	Ensemble, fixed weights	-3.05	0.10
Unibin log score	Season peak percentage	Ensemble, uniform weights	-3.16	0.08
Unibin log score	Season peak percentage	Delta density, Markovian	-3.28	0.16
Unibin log score	Season peak percentage	Delta density, extended (EDD)	-3.37	0.17
Unibin log score	Season peak percentage	ILINet + empirical futures	-5.18	0.23
Unibin log score	Season peak percentage	Empirical Bayes A (EBA)	-4.44	0.20
Unibin log score	Season peak percentage	Empirical Bayes B	-4.79	0.22
Unibin log score	Season peak percentage	Empirical Bayes C	-4.26	0.21
Unibin log score	Season peak percentage	BR, degenerate	-6.37	0.19
Unibin log score	Season peak percentage	BR, residual density	-3.98	0.17
Unibin log score	Season peak percentage	Targets, empirical	-8.12	0.41
Unibin log score	Season peak percentage	Targets, conditional density	-4.49	0.13
Unibin log score	Season peak percentage	Targets, uniform (UNIF)	-4.88	0.00
Unibin log score	Season peak percentage	AWE improvement on EDD	0.33	0.09
Unibin log score	Season peak percentage	EDD improvement on EBA	1.07	0.10
Unibin log score	Season peak percentage	EBA improvement on UNIF	0.44	0.20
Unibin log score	1 wk ahead	Ensemble, adaptive weights (AWE)	-2.65	0.05
Unibin log score	1 wk ahead	Ensemble, fixed weights	-2.68	0.04
Unibin log score	1 wk ahead	Ensemble, uniform weights	-2.91	0.04
Unibin log score	1 wk ahead	Delta density, Markovian	-2.94	0.07
Unibin log score	1 wk ahead	Delta density, extended (EDD)	-2.70	0.05

Unibin log score	1 wk ahead	ILINet + empirical futures	-6.48	0.11
Unibin log score	1 wk ahead	Empirical Bayes A (EBA)	-3.00	0.07
Unibin log score	1 wk ahead	Empirical Bayes B	-3.57	0.07
Unibin log score	1 wk ahead	Empirical Bayes C	-3.37	0.08
Unibin log score	1 wk ahead	BR, degenerate	-7.88	0.12
Unibin log score	1 wk ahead	BR, residual density	-3.09	0.06
Unibin log score	1 wk ahead	Targets, empirical	-6.48	0.11
Unibin log score	1 wk ahead	Targets, conditional density	-3.35	0.07
Unibin log score	1 wk ahead	Targets, uniform (UNIF)	-4.88	0.00
Unibin log score	1 wk ahead	AWE improvement on EDD	0.05	0.02
Unibin log score	1 wk ahead	EDD improvement on EBA	0.30	0.04
Unibin log score	1 wk ahead	EBA improvement on UNIF	1.87	0.07
Unibin log score	2 wk ahead	Ensemble, adaptive weights (AWE)	-2.84	0.05
Unibin log score	2 wk ahead	Ensemble, fixed weights	-2.88	0.04
Unibin log score	2 wk ahead	Ensemble, uniform weights	-3.07	0.04
Unibin log score	2 wk ahead	Delta density, Markovian	-3.09	0.06
Unibin log score	2 wk ahead	Delta density, extended (EDD)	-2.89	0.05
Unibin log score	2 wk ahead	ILINet + empirical futures	-6.51	0.11
Unibin log score	2 wk ahead	Empirical Bayes A (EBA)	-3.31	0.07
Unibin log score	2 wk ahead	Empirical Bayes B	-3.89	0.07
Unibin log score	2 wk ahead	Empirical Bayes C	-3.71	0.08
Unibin log score	2 wk ahead	BR, degenerate	-8.29	0.12
Unibin log score	2 wk ahead	BR, residual density	-3.24	0.06
Unibin log score	2 wk ahead	Targets, empirical	-6.51	0.11
Unibin log score	2 wk ahead	Targets, conditional density	-3.47	0.07
Unibin log score	2 wk ahead	Targets, uniform (UNIF)	-4.88	0.00
Unibin log score	2 wk ahead	AWE improvement on EDD	0.05	0.01
Unibin log score	2 wk ahead	EDD improvement on EBA	0.42	0.05
Unibin log score	2 wk ahead	EBA improvement on UNIF	1.57	0.07
Unibin log score	3 wk ahead	Ensemble, adaptive weights (AWE)	-2.99	0.04
Unibin log score	3 wk ahead	Ensemble, fixed weights	-3.01	0.04
Unibin log score	3 wk ahead	Ensemble, uniform weights	-3.18	0.04
Unibin log score	3 wk ahead	Delta density, Markovian	-3.19	0.06
Unibin log score	3 wk ahead	Delta density, extended (EDD)	-3.05	0.04
Unibin log score	3 wk ahead	ILINet + empirical futures	-6.54	0.11
Unibin log score	3 wk ahead	Empirical Bayes A (EBA)	-3.56	0.08
Unibin log score	3 wk ahead	Empirical Bayes B	-4.13	0.08
Unibin log score	3 wk ahead	Empirical Bayes C	-3.91	0.09
Unibin log score	3 wk ahead	BR, degenerate	-8.50	0.12
Unibin log score	3 wk ahead	BR, residual density	-3.35	0.05
Unibin log score	3 wk ahead	Targets, empirical	-6.54	0.11
Unibin log score	3 wk ahead	Targets, conditional density	-3.57	0.07
Unibin log score	3 wk ahead	Targets, uniform (UNIF)	-4.88	0.00
Unibin log score	3 wk ahead	AWE improvement on EDD	0.06	0.02
Unibin log score	3 wk ahead	EDD improvement on EBA	0.51	0.06
Unibin log score	3 wk ahead	EBA improvement on UNIF	1.32	0.08
Unibin log score	4 wk ahead	Ensemble, adaptive weights (AWE)	-3.10	0.04
Unibin log score	4 wk ahead	Ensemble, fixed weights	-3.12	0.04
Unibin log score	4 wk ahead	Ensemble, uniform weights	-3.28	0.04
Unibin log score	4 wk ahead	Delta density, Markovian	-3.29	0.05
Unibin log score	4 wk ahead	Delta density, extended (EDD)	-3.16	0.04
Unibin log score	4 wk ahead	ILINet + empirical futures	-6.57	0.11
Unibin log score	4 wk ahead	Empirical Bayes A (EBA)	-3.76	0.08
Unibin log score	4 wk ahead	Empirical Bayes B	-4.33	0.09
Unibin log score	4 wk ahead	Empirical Bayes C	-4.10	0.10
Unibin log score	4 wk ahead	BR, degenerate	-8.69	0.10
Unibin log score	4 wk ahead	BR, residual density	-3.41	0.05
Unibin log score	4 wk ahead	Targets, empirical	-6.57	0.11
Unibin log score	4 wk ahead	Targets, conditional density	-3.63	0.07
Unibin log score	4 wk ahead	Targets, uniform (UNIF)	-4.88	0.00

Unibin log score	4 wk ahead	AWE improvement on EDD	0.06	0.02
Unibin log score	4 wk ahead	EDD improvement on EBA	0.60	0.07
Unibin log score	4 wk ahead	EBA improvement on UNIF	1.12	0.08
Unibin log score	Overall	Ensemble, adaptive weights (AWE)	-2.53	0.04
Unibin log score	Overall	Ensemble, fixed weights	-2.55	0.04
Unibin log score	Overall	Ensemble, uniform weights	-2.72	0.04
Unibin log score	Overall	Delta density, Markovian	-2.74	0.06
Unibin log score	Overall	Delta density, extended (EDD)	-2.65	0.06
Unibin log score	Overall	ILINet + empirical futures	-5.25	0.11
Unibin log score	Overall	Empirical Bayes A (EBA)	-3.24	0.08
Unibin log score	Overall	Empirical Bayes B	-3.66	0.09
Unibin log score	Overall	Empirical Bayes C	-3.46	0.11
Unibin log score	Overall	BR, degenerate	-6.93	0.11
Unibin log score	Overall	BR, residual density	-3.11	0.06
Unibin log score	Overall	Targets, empirical	-6.41	0.15
Unibin log score	Overall	Targets, conditional density	-3.62	0.08
Unibin log score	Overall	Targets, uniform (UNIF)	-4.49	0.00
Unibin log score	Overall	AWE improvement on EDD	0.11	0.02
Unibin log score	Overall	EDD improvement on EBA	0.59	0.06
Unibin log score	Overall	EBA improvement on UNIF	1.25	0.08
