# Supplementary material for: Nonmechanistic forecasts of seasonal influenza with iterative one-week-ahead distributions
Source: PLoS Comput Biol. 2018 Jun 15;14(6):e1006134. doi: 10.1371/journal.pcbi.1006134 (PMC6034894; doi:10.1371/journal.pcbi.1006134)
Supplement: S5 Appendix — (PDF) [file pcbi.1006134.s017.pdf]

Log of changes to Delphi-Stat throughout the 2015/2016 season and for cross-validation analysis

Initial description (2015 EW42)

The Delphi-Stat system is an ensemble of several baselines and statistical forecasting methods. Its forecasts are a linear combination of the forecasts of these individual systems, with a separate set of coefficients determined for each epi week, geographical area (nation + 10 HHS regions), metric (MAE or log score), and target. The methods are outlined below. Note that the term "past epiweeks" refers to a set of epi week numbers in any season — specifically, epi weeks 21 up to the forecast week; "future epiweeks" is used in a similar fashion. (Nonnegative coefficients summing to 1 are calculated for point predictions using constrained LAD regression (implemented using the linear programming package `lpSolve` [49]), and for distributional predictions with the degenerate EM algorithm [48].)

- **Empirical prior:** ignores all data from the current season, and considers each training season — 2003/2004 to 2014/2015, excluding the pandemic — as equally likely to reoccur.
- **Pinned baseline:** uses the available observations for the current season for previous epi-weeks; for future epi weeks, each training curve is considered equally likely to reoccur.
- **Basis regression:**
    1. Aligns training curves with the current season by shifting in time and scaling weighted ILI values until the maximum of each training curve in past epiweeks is the same as that of the current season. (Scaling is performed only above the CDC baseline; if a curve is entirely below the CDC baseline, it is not scaled at all.)
    2. Fits a smooth curve to the observed data in past epiweeks and the mean of the aligned training curves in future epiweeks. (The smooth curve is a spline: specifically, a linear combination of B-splines selected with elastic net using the glmnet package [SAE1], with a tradeoff penalty between the importance of matching past and future epiweeks.)
    3. Uses observations from the current season in past epiweeks; considers this single curve as the only possibility for future weeks.
- **Basis regression with noise:**
    1. Generates the spline curve above.
    2. Considers the spline as estimating the change in weighted ILI from one week to the next; for each epi week, estimates the distribution of errors at that epi week using the training curves. (Distributions

are estimated using weighted kernel density estimation: when adding noise to a simulated 2015/2016 curve at some future epiweek, training curves that more closely resemble the simulated curve in previous epiweeks contribute more to the result.)

3. Generates many simulated 2015/2016 curves by taking the observations from the current season so far, and at each week, adding the estimated change from the spline curve, then drawing a value from the estimated error distribution.

- **Time-parameterized weighted kernel density estimation:** Follows the same process as the basis regression with noise; however, it directly estimates the distribution of changes in weighted ILI values, rather than the corresponding distribution of errors in the spline estimate.
- **Empirical Bayes:** We use the procedure described in this document [21], with a few modifications: a smoothed (trend-filtered [SAE2]) curve is never paired with a noise estimate from another smoothed curve, scaling and shifting is performed only in small amounts resulting in "local" transformations, an additional component is added to the likelihood to encourage reasonable predictions at future weeks (by penalizing simulated curves if they deviate too much from all of the training curves), and incorporating a random inflation in the noise parameter to prevent forecast "overconfidence".
- **Uniform prior:** Considers each cell in the spreadsheet to be equally likely. (This component only produces distributional forecasts.) Additional weight is added to this component after the coefficients for each method are determined via cross-validation to prevent any 0 or near-0 probability forecasts.

Changes, 2015 EW43

- **Mixing coefficients between methods:** a set of weights for each of the forecasting methods is determined for each epi week, metric (MAE or log score), and target, but are tied across areas (nation + 10 HHS regions); thus, any method will receive the same weight in all areas (for the same epi week, metric, and target). For distributional forecasts, the weight assigned to the uniform distribution is increased by approximately 2.5% (based on the rule of three), and weight taken away evenly from all methods to make the weights again sum to 1. This is accomplished by changing the RelevanceWeight function from

$$\text{RelevanceWeight}(s,l,t,i,e;s',l',t',i',e') = \begin{cases} 1, & l=l',\ t=t',\ i=i',\ e=e' \\ 0, & \text{otherwise} \end{cases}$$

to

$$\text{RelevanceWeight}(s,l,t,i,e;s',l',t',i',e') = \begin{cases} 1, & t=t',\ i=i',\ e=e' \\ 0, & \text{otherwise,} \end{cases}$$

and setting μ as described in the main text. These changes motivated on two hypotheses:

  - The previous weight vector calculations, which previously only considered 11 training instances at a time (one per season from 2003/2004 to 2014/2015, excluding 2009/2010), were based on much too little data, and considering training instances from other locations would be beneficial (even though training data from other locations seems less relevant than training data from the same location).
  - The μ value from the rule of three will be more appropriate than an μ value selected to ensure an arbitrary minimum log score value, and will automatically update based on the amount of training data available.

- **New method added to ensemble:** direct target density estimation: uses the same weighted kernel density estimation approach as two existing methods to directly forecast each of the targets without constructing, rather than constructing a distribution of flu curves and extracting the target values from these curves. Adjustments to the output are made so that all predicted possible values are integers when appropriate and lie in the correct range.

Changes and clarifications, 2015 EW44

- **New method added to ensemble:** modified time-weighted kernel density estimation: this version changes the weighting criteria used for matching simulated data for this year to past seasons; attempts to make simulated trajectories more closely resemble past seasons' data; and considers a wider range of past data. When constructing trajectories, this version weights past seasons based on the previous weeks wILI value; the sum of previous wILI values in the season; a weighted sum of wILI values stressing more recent weeks; and a weighted sum of the week-to-week changes in wILI stressing more recent times. With low probability, these weights are ignored and a random change in wILI is selected from historical data. The simulated data values are also pushed towards randomly selected historical data by a small amount. When simulating data at epi week t, instead of just looking at other seasons at week t, also considers nearby weeks, unless t is a time near the end of year holidays.
- **Clarification:** older kernel density estimation method, direct target density estimation: only weight data based on the previous wILI value.

Changes, 2015 EW46

- **Backfill forecasting:** we now use backfill forecasting in combination with almost all of the forecasting methods in the Delphi-Stat ensemble. For each nonfinal wILI value in the current season, we estimate a distribution

for its final revised value. The distribution is based on historical revisions of wILI with the same lag (e.g., the latest measurement vs. the second most recent measurement), and is formed using weighted kernel density estimation, with weights depending on the epiweek to which the measurement corresponds, and the nonfinal wILI value itself.

Changes, 2016 EW03

Another statistical method has been added to the Delphi-Stat ensemble:

- **Target forecast:** We use an additive model to create predictions that are target specific using the past 3 values observed.

Changes, for cross-validation analysis

- **Changes to ensemble weight training data:** ensemble weights are selected using cross-validation component forecasts based on the version of test season data that would have been available at the forecast time, rather than ground truth; since regional back issues are available starting only in late 2009, cross-validation analysis is performed on seasons 2010/2011 to 2015/2016 as described in the main text.

- **Changes to RelevanceWeight function:** the RelevanceWeight function still seems like it will lead to ensemble weight vectors based on too little training data, especially considering the reduction in the number of training seasons, so we use the RelevanceWeight function specified in the text, which considers cross-validation component evaluations from forecast weeks within 4 weeks of t when setting weights for forecast week t (chosen to include many additional weeks while keeping early-season evaluations from influencing late-season weights, and late-season evaluations from influencing early-season weights).

- **Changes to methods in ensemble:** the additive model was removed from the ensemble to ease system maintenance, and the two Empirical Bayes variants were added to compare cross-validation forecast behavior and potentially improve the ensemble performance.

Supplementary references

[SAE1] Friedman J, Hastie T, Tibshirani R. Regularization Paths for Generalized Linear Models via Coordinate Descent. Journal of Statistical Software. 2010;33(1):1–22.

[SAE2] Tibshirani RJ. Adaptive piecewise polynomial estimation via trend filtering. The Annals of Statistics. 2014;42(1):285–323.
